# Multidisciplinary Approaches Identify Compounds that Bind to Human ACE2 or SARS-CoV-2 Spike Protein as Candidates to Block SARS-CoV-2–ACE2 Receptor Interactions

**DOI:** 10.1128/mBio.03681-20

**Published:** 2021-03-30

**Authors:** Christopher J. Day, Benjamin Bailly, Patrice Guillon, Larissa Dirr, Freda E.-C. Jen, Belinda L. Spillings, Johnson Mak, Mark von Itzstein, Thomas Haselhorst, Michael P. Jennings

**Affiliations:** a Institute for Glycomics, Griffith University, Gold Coast Campus, Gold Coast, QLD, Australia; Virginia Tech; Virginia Polytechnic Institute and State University

**Keywords:** ACE2, SARS-CoV-2, drug screening

## Abstract

Severe acute respiratory syndrome coronavirus 2 (SARS-CoV-2) is a recently emerged virus that causes coronavirus infectious disease 2019 (COVID-19). SARS-CoV-2 spike protein, like SARS-CoV-1, uses the angiotensin converting enzyme 2 (ACE2) as a cellular receptor to initiate infection. Compounds that interfere with the SARS-CoV-2 spike protein receptor binding domain protein (RBD)-ACE2 receptor interaction may function as entry inhibitors. Here, we used a dual strategy of molecular docking and surface plasmon resonance (SPR) screening of compound libraries to identify those that bind to human ACE2 or the SARS-CoV-2 spike protein receptor binding domain (RBD). Molecular modeling screening interrogated 57,641 compounds and focused on the region of ACE2 that is engaged by RBD of the SARS-CoV-2 spike glycoprotein and vice versa. SPR screening used immobilized human ACE2 and SARS-CoV-2 Spike protein to evaluate the binding of these proteins to a library of 3,141 compounds. These combined screens identified compounds from these libraries that bind at *K_D_* (equilibrium dissociation constant) <3 μM affinity to their respective targets, 17 for ACE2 and 6 for SARS-CoV-2 RBD. Twelve ACE2 binders and six of the RBD binders compete with the RBD-ACE2 interaction in an SPR-based competition assay. These compounds included registered drugs and dyes used in biomedical applications. A Vero-E6 cell-based SARS-CoV-2 infection assay was used to evaluate infection blockade by candidate entry inhibitors. Three compounds demonstrated dose-dependent antiviral *in vitro* potency—Evans blue, sodium lifitegrast, and lumacaftor. This study has identified potential drugs for repurposing as SARS-CoV-2 entry inhibitors or as chemical scaffolds for drug development.

## Abstract (precis)

SARS-CoV-2, the causative agent of COVID-19, has caused more than 60 million cases worldwide with almost 1.5 million deaths as of November 2020. Repurposing existing drugs is the most rapid path to clinical intervention for emerging diseases.

## INTRODUCTION

Severe acute respiratory syndrome coronavirus 2 (SARS-CoV-2) is a recently emerged virus that causes an often-fatal respiratory disease, COVID-19. The current pandemic caused by SARS-CoV-2 is a health emergency that requires the development of new vaccines and drugs to prevent or treat this disease. Most antiviral drug strategies target viral proteins or host factors required for intracellular replicative processes. Inhibiting viral entry into host cells via blocking access to cell surface viral receptors can also be a successful strategy, the best example being the entry inhibitor drug maraviroc, which binds to the human immunodeficiency virus 1 (HIV-1) coreceptor CCR5 to block infection (1). The entry-blocking approach has been targeted with therapeutic antibodies (2); however, this approach targets the SARS-CoV-2 spike protein, whereas drugs have the potential to also target the host receptor for the virus. SARS-CoV-2 is closely related to severe acute respiratory syndrome coronavirus (SARS-CoV-1) (3), and recent studies have demonstrated that the SARS-CoV-2 spike protein, like SARS-CoV-1, uses the angiotensin converting enzyme 2 (ACE2) as a cellular receptor to engage with host cells (4). SARS-CoV-2 engages the ACE2 receptor with higher-affinity binding than SARS-CoV-1 (5). Upon binding to ACE2, the SARS-CoV-2 spike protein needs to be activated by cellular proteases, such as TMPRSS2, to initiate the spike-mediated fusion of the viral envelope with the host-cell membrane (4, 6). This process may be facilitated by the preactivation of the spike protein by furin, which reduces the dependence of SARS-CoV-2 on TMPRSS2 for entry. Independently of this secondary mechanism for entry, the SARS-CoV-2 spike receptor binding domain (RBD) was found to have a higher affinity for ACE2 than the SARS-CoV spike RBD, making it an ideal target to block the attachment of virus to host cells for drug discovery (4, 6). Repurposing existing drugs is the most rapid path to clinical intervention for emerging diseases. In the context of SARS-CoV-2 research, several studies have used high-throughput target-based (i.e., against spike, M^pro^) or phenotypic screens, as well as *in silico* studies, to identify inhibitors of SARS-CoV-2 infection. (4, 7–24). Here, we apply a similar screening strategy that was employed in one of our recent studies, where we used a surface plasmon resonance (SPR)-based high-throughput biophysical screen to identify drugs that bind to human complement receptor 3 as a host-receptor-blocking strategy to prevent bacterial infection (25). With this method, used in combination with molecular docking screening and *in vitro* antiviral screening approaches, we identify compounds that bind to ACE2 or to the SARS-CoV-2 spike protein RBD and that block SARS-CoV-2 *in vitro* infection.

## RESULTS

### Molecular docking screening of drugs that bind to ACE2.

**FIG 1:**
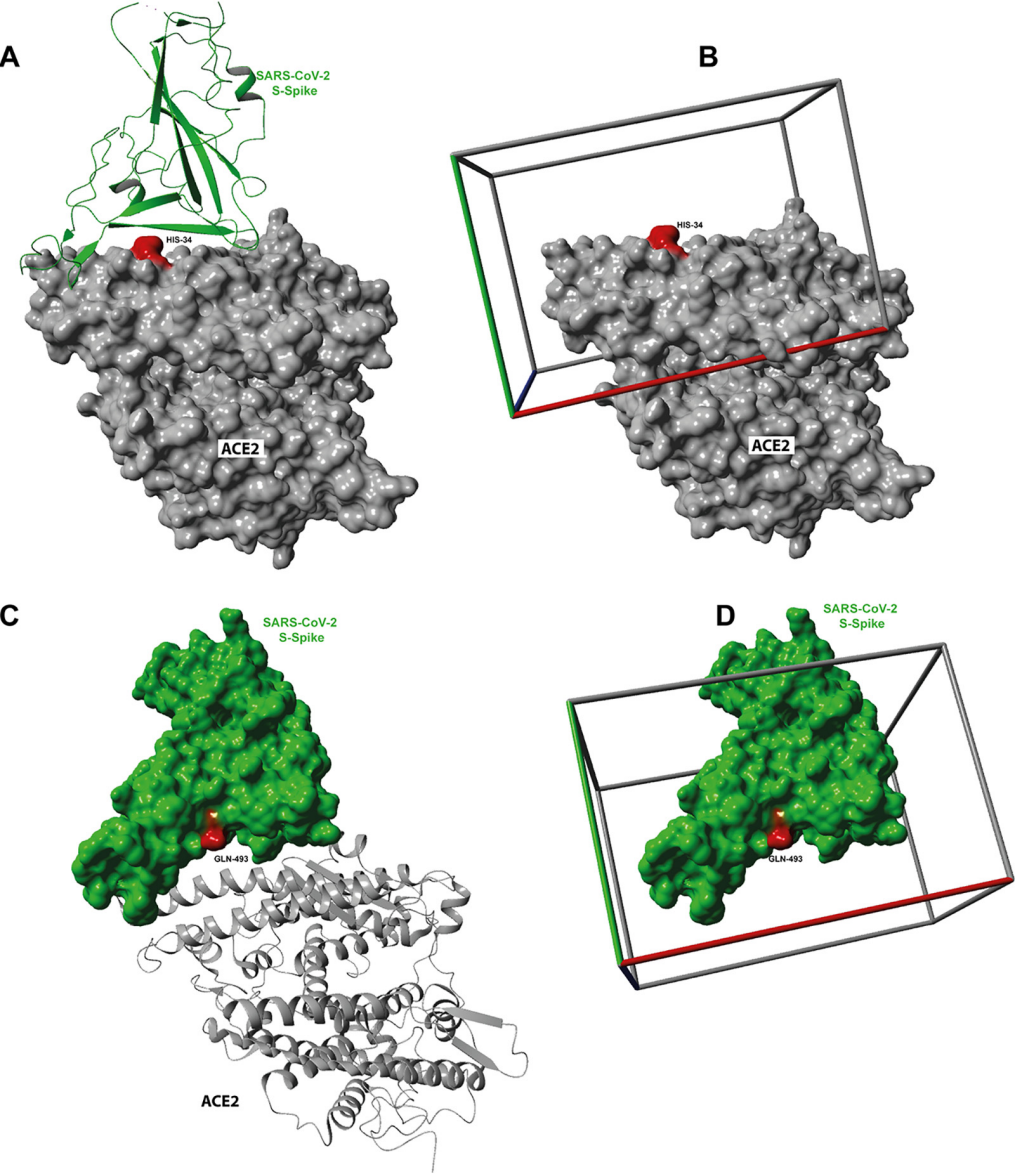
The structure of the human ACE2-S1 domain (RBD) of the SARS-CoV spike protein initially used to select regions for the molecular docking screen. The structure was determined using PDB 2AJF (53). (A) SARS-CoV S spike protein bound to ACE2 with marked ACE2 active site and SARS-CoV S protein interacting site. (B) For molecular docking screening experiments, SARS-CoV S spike protein was removed, and a rectangular box (50 Å by 60 Å by 40 Å) was centered around HIS-34. (C) For molecular docking screening experiments, the structure of the SARS-CoV-2 chimeric receptor-binding domain SARS-CoV-2 (PDB 6VW1) was used with 2.68-Å resolution (28). (D) The human ACE2 protein was removed, and a rectangular box (50 Å by 60 Å by 50 Å) was centered around GLN-493.

A library of 57,641 molecules were docked with 12 runs each into the SARS-CoV-2 spike protein interacting site of ACE2 with the position of the molecular docking screening box centered at HIS-34 (Fig. 1). Docked conformation of ligands that did not interact with the main amino acids of ACE2 that mediate binding with SARS-CoV-2 spike protein RBD (53) were eliminated. The predicted binding affinities of the best poses for each binding ligand are shown in Fig. S1A to Q, along with a molecular representation of the interactions of each compound with human ACE2. The ligands with the highest predicted affinities for ACE2 are an Evans blue mimetic (Cas no. 303106-55-0 *K_D_* [equilibrium dissociation constant], 124 nM) and ledipasvir (*K_D_*, 232 nM), a drug that is used for the treatment of hepatitis C (27).

10.1128/mBio.03681-20.1 FIG S1 Molecular docking of the compounds identified for ACE2. Docked compounds in the ACE2 crystal structure and the predicted binding affinity. (A) Ledipasvir; (B) venetoclax; (C) CID 3110549; (D) irinotecan; (E) digitoxin; (F) CID 16455811; (G) gedatolisib; (H) digoxin; (I) velpatasvir; (J) radotinib; (K) zotarolimus; (L) caspofungin; (M) acalabrutinib; (N) epigallocatechin-3-gallate; (O) Evans blue mimetic; (PP) levodopa; (Q) Chicago sky blue. Download FIG S1, PDF file, 1.2 MB. Copyright © 2021 Day et al. 2021 Day et al. https://creativecommons.org/licenses/by/4.0/ This content is distributed under the terms of the Creative Commons Attribution 4.0 International license.

### Surface plasmon resonance screening for drugs that bind to ACE2.

**FIG 2:**
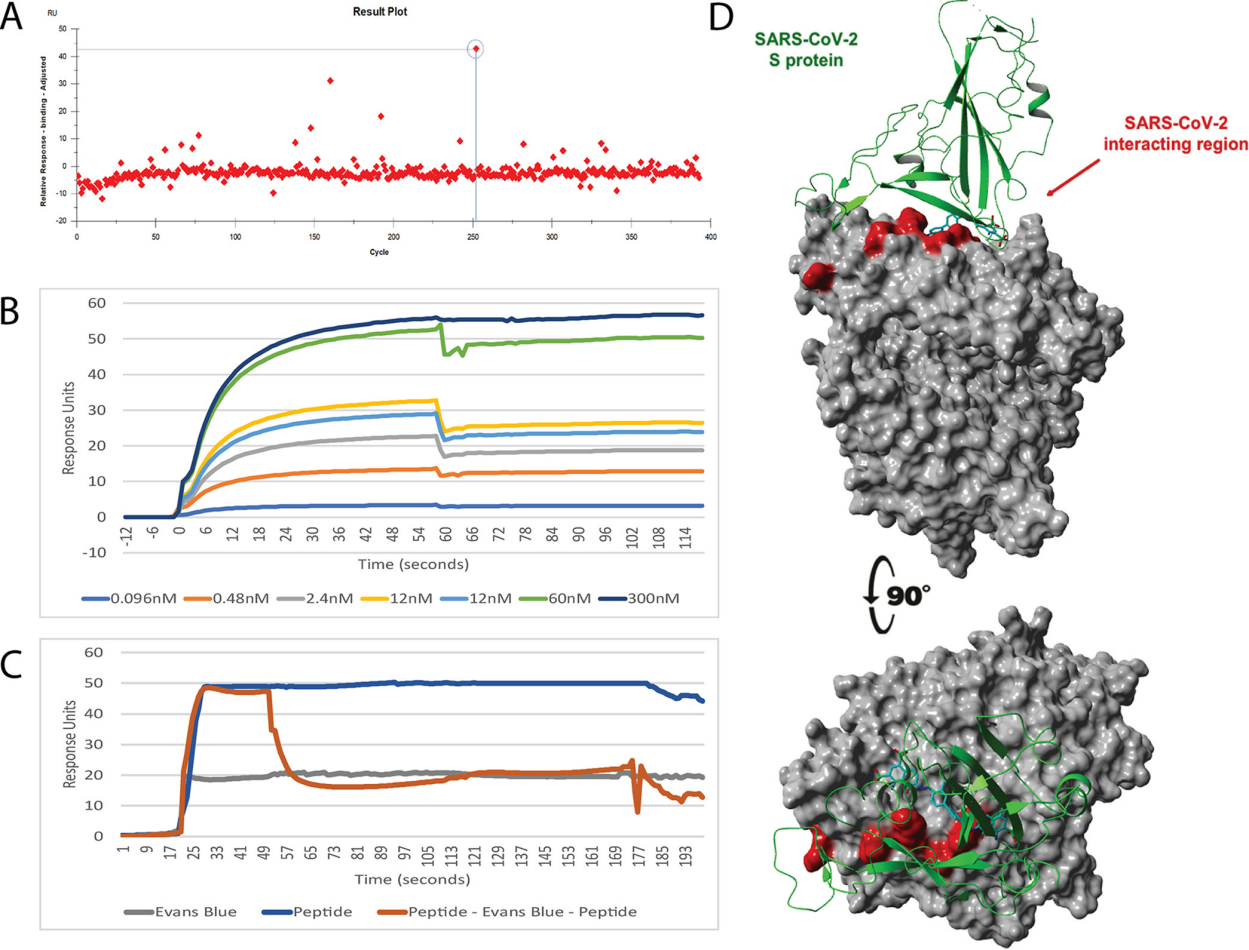
SPR analysis and molecular dynamics analysis of the ACE2 binding compound Evans blue. (A) Graphical representation showing Evans blue identification in the SPR screen. The circled spot is Evans blue (Compounds Australia sample no. SN01005402; molecular weight, 962 g/mol). (B) Sensor gram of the multicycle interaction between Evans blue and ACE2. (C) Competition assay between Evans blue and the RBD-1 peptide for ACE2. The blue line is the peptide injection for the whole injection (injections A, B, and A)–peptide control. Gray line is the Evans blue (injections A, B, and A)–compound-only control. Orange is the peptide (Both A injections) and compound (Evans blue; injection B)–competition curve. (D) Molecular docking results of the highest-affinity ligand Evans blue (also called T-1824) in complex with human ACE2. Evans blue has a *K_D_* of 1.6 nM by SPR. Shown in green is the secondary ribbon structure of the S1 RBD of the SARS-CoV-2.

A library of 3,141 compounds was screened for binding to the human ACE2 by SPR, as described previously (25). Initial screening identified compounds that bound ACE2, with positives defined as those binding at least 5 response units above the negative control at a concentration of 1 μM (Fig. 2A and Fig. S2). Each unique ACE2 binding hit that had not already been identified by the molecular docking screen strategy (above) was analyzed by molecular modeling to determine those drugs that are predicted to bind at the ACE2–SARS-CoV-2 RBD interface. The compounds identified in the molecular docking screening (Table 1), if readily available, and the SPR screened hits were all analyzed by SPR to measure the binding affinity for recombinant human ACE2 (Table 1). The SPR-determined affinities were in the same hierarchy as the predicted affinities determined by molecular docking (Table 1 and Fig. 2B).

**TABLE 1:** ACE2 binding compounds determined by molecular docking and SPR screening of drug libraries^*a*^

Name	*K_D_* (nM)^*c*^	1:1 competition between compound vs RBD-mimic1 peptide at 1 μM	Compound information	SARS-CoV-2-related studies
Evans blue^*b*^	1.63 ± 0.08	99.8% ± 3.84	Dye used in some biomedical applications (48, 61)	None
Levodopa^*b*^	13.6 ± 0.97	36.4% ± 1.62	Treatment for Parkinson’s disease (62)	None
Epigallocatechin-3-gallate^*b*^	13.7 ± 1.90	28.9% ± 1.43	A catechin from Camellia sinensis	Clinical trial (26)
Velpatasvir	24.9 ± 4.24	61.4% ± 0.34	Treatment of hepatitis C (NS5A inhibitor) (63)	Protease inhibitor trials (12)
Acalabrutinib	25.7 ± 0.91	77.4% ± 3.9	Chemotherapeutic drug (c-BKT inhibitor) (64)	IL-6 inhibitor clinical trial (39)
Venetoclax	290 ± 31.1	66.3% ± 4.1	Chemotherapeutic drug (BCL-2 inhibitor) (40)	None
Chicago sky blue^*b*^	349 ± 29.8	2.47% ± 0.24	Dye, pharmacologically active (38)	None
Ledipasvir	417 ± 50.7	79.4% ± 2.1	Treatment of hepatitis C (NS5A inhibitor) (65)	Protease inhibitor trails (39)
Irinotecan	825 ± 112	100% ± 6.36	Chemotherapeutic drug (topoisomerase inhibitor) (66)	None
Digitoxin	1480 ± 87.6	25.1% ± 2.42	Cardiac glycoside for treating atrial fibrillation (67)	None
Digoxin	1254 ± 97.2	27.6% ± 1.39	Cardiac glycoside for treating atrial fibrillation (67)	None
Zotarolimus	2764 ± 178	69.4% ± 3.97	Immunosuppressant use in cardiac stents (32)	None
CID 3110549	ND	ND	Compound (see PubChem)	None
CID 16455811	ND	ND	Compound (see PubChem)	None
Gedatolisib	ND	ND	Chemotherapeutic drug (mTOR/PI3K inhibitor) (68)	None
Radotinib	ND	ND	Chemotherapeutic drug (c-Abl inhibitor) (69)	Antiviral activity (70)
Caspofungin	ND	ND	Antifungal drug (72)	None
RBD-mimic1 peptide	13.7 ± 2.4	NA		
RBD-mimic2 peptide	347 ± 102	NA		

a Compounds identified by molecular docking screening using AutoDock Vina of 57,641 compounds from various sources against the SARS-CoV-2 spike protein interacting site (RBD) with ACE2 (see Fig. 1 and 2 and Fig. S1 to S5). ND, not done; NA, not applicable. *K_D_* ± one standard deviation of duplicate technical repeats on two separate biological repeats.

b Not detected in original molecular docking screen.

c *K_D_* determined by SPR.

10.1128/mBio.03681-20.2 FIG S2 SPR result plots for the identification of ACE2 binding compounds. Graphical representation showing compound identification in the SPR screen. (A) Evans blue; (B) levodopa; (C) epigallocatechin-3-gallate; (D) Chicago sky blue. Download FIG S2, PDF file, 0.07 MB. Copyright © 2021 Day et al. 2021 Day et al. https://creativecommons.org/licenses/by/4.0/ This content is distributed under the terms of the Creative Commons Attribution 4.0 International license.

### Competition studies between drugs and a SARS-CoV-2 RBD peptide mimic, RBD-mimic1, for ACE2 binding.

To examine the ability of each drug to block the SARS-CoV-2 spike protein from binding ACE2, we conducted an SPR competition study using immobilized ACE2. At the time of this screening, SARS-CoV-2 spike protein was not available for our studies, so we designed peptides containing all the key ACE2-interacting residues of the RBD, based in the model SARS-CoV-2 spike protein RBD-ACE2 complex (PDB 6VW1; see Fig. 2A). SPR studies showed that a peptide, RBD-mimic 1 (NCYFPLQSYGFQPTNGV), binds recombinant human ACE2 with high affinity (*K_D_*, 13.7 ± 2.4 nM; Table 1) and essentially recapitulates the recently reported ∼15-nM *K_D_* for the SARS-CoV-2 spike protein-ACE2 interaction (5). A second peptide, RBD-mimic 2, had a lower-affinity *K_D_* of 347.2 ± 102 nM and was not used in further studies (Table 1). Five of the compounds identified in the *in silico* screening could not be sourced for further evaluations (NDs in *K_D_* column in Table 1).

The competition analysis revealed that 11 of the 12 compounds that were tested can fully or partially inhibit RBD-mimic1 peptide-ACE2 interaction (Table 1), with seven compounds blocking greater than 60% (Fig. S3). Two of the tested compounds, Evans blue (Fig. 2C) and Irinotecan, showed complete inhibition of the RBD-mimic1 peptide-ACE2 interaction at a 1:1 ratio at 1 μM (Table 1; Fig. S3). Subsequent competition assays using recombinant ACE2 and immobilized virus-like particles (VLPs) expressing SARS-CoV-2 spike protein showed the same pattern of blocking as the RBD-1 peptide-based assays shown in Table 1 (see Table S1).

10.1128/mBio.03681-20.3 FIG S3 SPR result plots for competition of identified compounds with the ACE2:RBD-spike peptide. Competition assay between compounds and the RBD-1 peptide for ACE2. The blue line is peptide injection for the whole injection (injections A, B and A) peptide control. Gray is the compound (injections A, B and A)–compound only control. Orange is the peptide (both A injections) and compound (injection B) competition curve. (A) Irinotecan; (B) velpatasvir; (C) venetoclax; (D) ledipasvir. Download FIG S3, PDF file, 0.3 MB. Copyright © 2021 Day et al. 2021 Day et al. https://creativecommons.org/licenses/by/4.0/ This content is distributed under the terms of the Creative Commons Attribution 4.0 International license.

10.1128/mBio.03681-20.6 TABLE S1 Competition of ACE2 binding compounds determined by molecular docking and SPR screening of drug libraries with immobilized virus-like particles (VLPs) expressing SARS-CoV2 spike protein Table S1, PDF file, 0.09 MB. Copyright © 2021 Day et al. 2021 Day et al. https://creativecommons.org/licenses/by/4.0/ This content is distributed under the terms of the Creative Commons Attribution 4.0 International license.

### Molecular docking and SPR screening of SARS CoV-2 spike protein.

**FIG 3:**
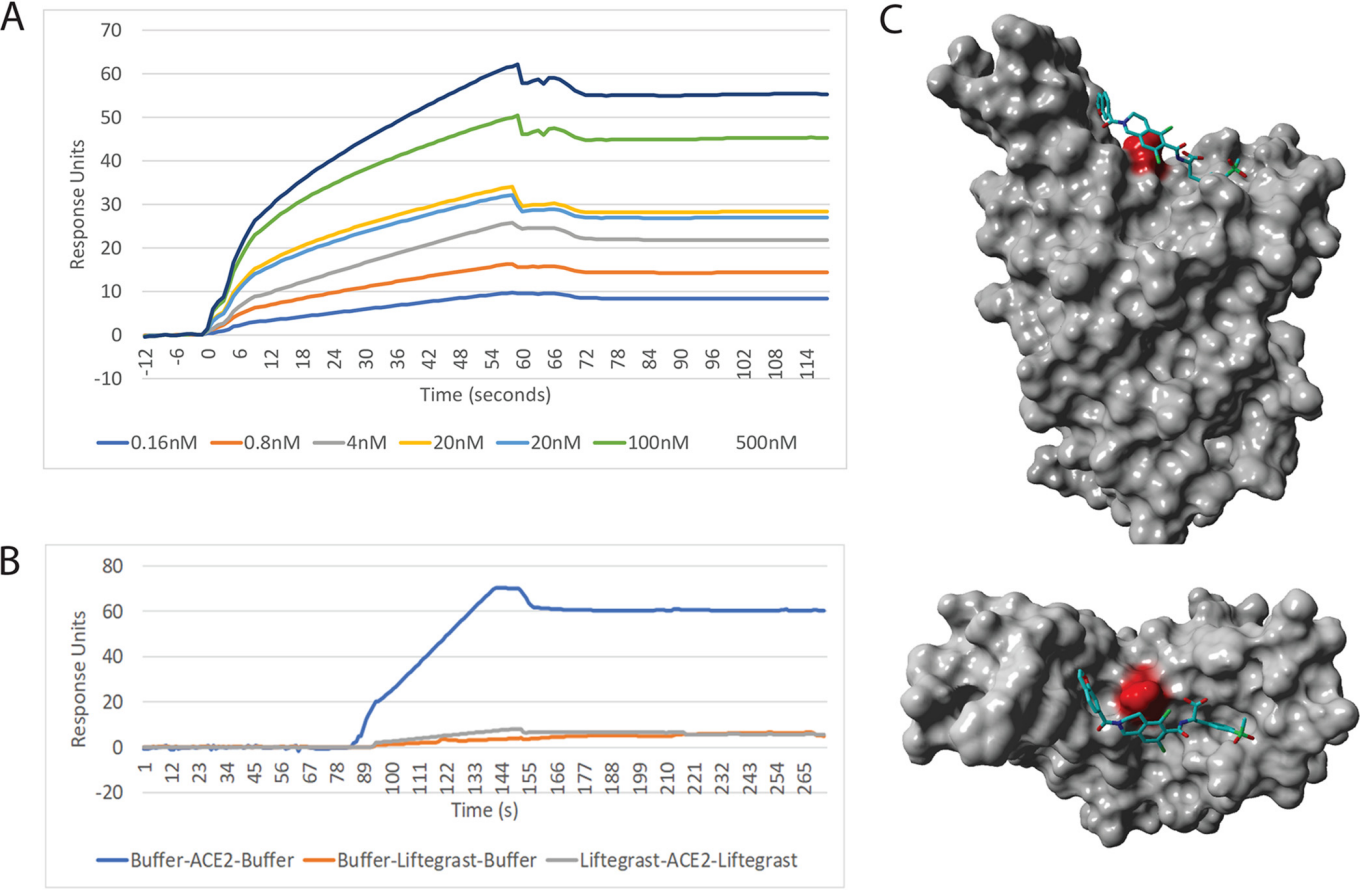
SPR analysis and molecular dynamics analysis of the CoV2 spike protein binding compound lifitegrast. (A) Sensor gram of the multicycle interaction between lifitegrast and CoV2 spike protein. (B) Competition assay between lifitegrast and the recombinant ACE2 protein for spike protein. (C) Molecular docking screening results of the highest-affinity ligand lifitegrast in complex with CoV2 spike protein.

The three-dimensional X-ray crystal structure of the SARS-CoV-2 chimeric receptor-binding domain complexed with its receptor human ACE2 was obtained from the protein databank (http://rcsb.org) (PDB 6VW1) (28). A rectangular box with dimensions 50 Å by 60 Å by 50 Å (*x*, *y*, and *z*) was centered at the amino acid SER-494 of the SARS-CoV-2 -spike protein coordinates (see Fig. 1 and 3). A total of 57,641 compounds were docked against SARS-CoV-2 spike protein with a total computing time of ∼7 days. This virtual molecular docking screen identified 8 compounds that bound with high theoretical affinity to the boxed region of the human SARS-CoV-2 spike protein (Table 2 and Fig. S4).

**TABLE 2:** SPR analysis affinity of identified compounds for SARS-CoV-2 Spike protein receptor binding domain^*a*^

Compound	*K_D_*	Blocking (%)	Compound information	SARS-CoV-2-related studies
Sodium lifitegrast	1.92 nM ± 0.08	99.8 ± 6.4	Used for treatment of keratoconjunctivitis sicca	Predicted CoV2 Nsp16 binder (14) and Nsp13 (21)
Cefpiramide	330 nM ± 14.7	81.9 ± 8.4	Broad-spectrum, cephalosporin,	Potential protease inhibitor (72)
Dactinomycin	455 nM ± 22.1	69.7 ± 1.8	Chemotherapy medication	Potential combo therapy (24) historic coronavirus inhibitor (31)
Simeprevir	819 nM ± 0.14	88.6 ± 7.2	Inhibitor of the hepatitis C virus	RBD binder with lumacaftor (20)
Lumacaftor (VX809)	1.51 μM ± 0.11	71.4 ± 3.1	Cystic fibrosis transmembrane conductance regulator (CFTR)	RBD binder with Simeprevir (20)
Evans blue	2.21 μM ± 0.14	78.2 ± 6.3	Dye used in some biomedical applications (48, 61)	None

a Compounds identified by molecular docking screening using AutoDock Vina of 57,641 compounds from various sources against the SARS-CoV-2 spike protein interacting site (RBD) with ACE2 (see Fig. 1 and 3 and Fig. S1 to S5). ND, not done. *K_D_* ± one standard deviation of duplicate technical repeats on two separate biological repeats.

10.1128/mBio.03681-20.4 FIG S4 Molecular docking of the compounds identified for SARS-CoV-2 spike protein. Docked compounds in the SARS-CoV-2 spike protein crystal structure and the predicted binding affinity. (A) Evans blue; (B) dactinomycin; (C) cefparmide; (D) lifitegrast; (E) lumacaftor (VX809). Download FIG S4, PDF file, 0.4 MB. Copyright © 2021 Day et al. 2021 Day et al. https://creativecommons.org/licenses/by/4.0/ This content is distributed under the terms of the Creative Commons Attribution 4.0 International license.

VLPs with and without the SARS CoV2 spike protein were tested for binding to the same library of 3,141 compounds by SPR analysis. Initial screening of the compounds at 1 μM identified three compounds, cefpiramide, dactinomycin, and Evans blue, binding to the VLP spike protein, all three of them overlapping the molecular docking screen (see Table 2). Only 6 of the 8 identified compounds could be sourced for a secondary screening using SPR to test for binding affinity and for the ability to block human ACE2-VLP spike protein interactions in direct competition SPR experiments (see Fig. S4 and Table 2). The highest-affinity compound identified for the SARS-CoV-2 spike protein was sodium lifitegrast with a *K_D_* of 1.92 nM. This drug can eliminate 99.8% of the RBD-ACE2 protein-protein interaction in SPR competition assays (Table 2, Fig. 3).

### Antiviral potency of drug hits against SARS-CoV-2 infection of Vero-E6 cells.

**FIG 4:**
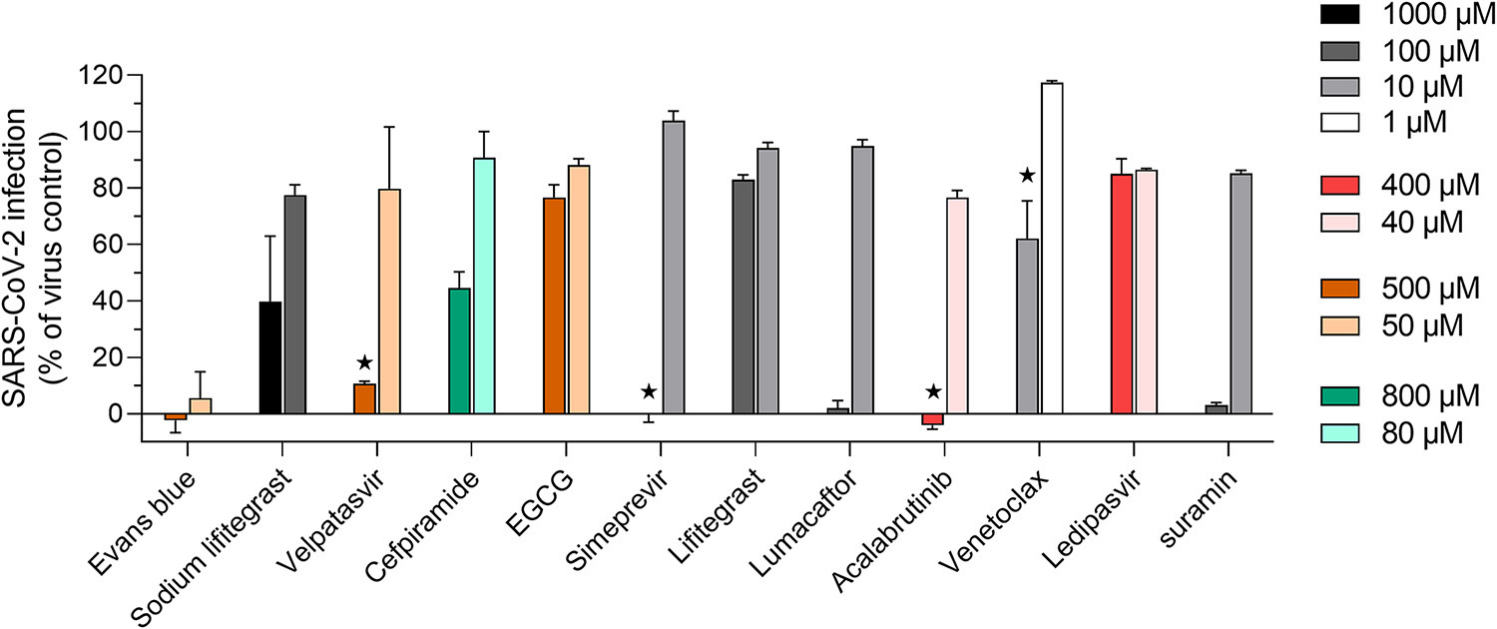
Screening of approved drugs against SARS-CoV-2 *in vitro* infection of Vero-E6 cells. Confluent Vero-E6 cells were incubated with virus and compound dilutions for 48 h at 37°C, after which infection was measured with *in situ* ELISA using a primary SARS-CoV-2 nucleocapsid antibody. Bars represent the average ± standard deviation (SD) of duplicate measurements. Black stars denote obvious compound-induced cytotoxicity.

Of the 22 compounds identified through *in silico* and SPR screening, 11 that showed blocking in SPR studies and that were not overtly toxic and did not have solubility issues were tested against SARS-CoV-2 infection of Vero-E6 cells (Fig. 4). Eleven compounds were not tested in the Vero-E6 cell assay due to known toxicity or insolubility in aqueous solution to the concentrations required. Out of the 11 compounds tested, 4 were found to be toxic at the highest concentration tested while inactive at lower concentrations—velpatasvir, simeprevir, acalabrutinib, and venetoclax. Four compounds were found to be active at the highest concentration tested with no apparent cytotoxicity—Evans blue, sodium lifitegrast, cefpiramide, and lumacaftor. Suramin, which was used as a positive control of inhibition, demonstrated 100% antiviral potency at 100 μM, as previously published (29).

Evans blue, sodium lifitegrast, cefpiramide, and lumacaftor were further evaluated in dose-response experiments to determine their *in vitro* potency (50% inhibitory concentration [IC_50_]) against SARS-CoV-2 infection as well as their toxicity toward Vero-E6 cells (50% cytotoxic concentration [CC_50_]). Despite being approved drugs, some compounds were found to be toxic in Vero-E6 cells during screening, which was conducted over a 48-h infection (Fig. 4). The dose-response experiments were therefore optimized for a 24-h infection to reduce the risks of Vero-E6. As shown in Table 3 (dose-response curves available in Fig. S5), Evans blue had an IC_50_ value of 28.1 ± 1.2 μM with no cytotoxicity at 1,000 μM, in the same order of magnitude as values obtained for the closely chemically related drug suramin (IC_50_, 46.2 ± 1.6 μM; CC_50_, >1,000 μM). The drug sodium lifitegrast could block SARS-CoV-2 *in vitro* infection with a potency of 1,295.3 ± 25.1 μM while remaining noncytotoxic at a concentration as high as 5 mM. Finally, lumacaftor had IC_50_ and CC_50_ values of 84 ± 3.7 μM and 314.5 ± 22.7 μM, respectively. Cefpiramide, however, was found to be inactive at 1.5 mM. In light of these results, Evans blue possessed the highest selectivity index in Vero-E6 cells of >35.6, against >21.6, >3.9, and 3.7 for suramin, sodium lifitegrast, and lumacaftor, respectively. All other compounds were found to be either inactive or cytotoxic to Vero E6 cells in dose-response experiments.

**TABLE 3:** *In vitro* potency of hits against SARS-CoV-2 infection and cytotoxicity toward Vero-E6 cells^*a*^

Compound	IC_50_ (μM)		CC_50_ (μM)		SI^*b*^	Screening data	*C_max_*^*c*^
	Mean ± SD	*n*	Mean ± SD	*n*			
Evans blue	28.1 ± 1.2	3	>1,000	3	>35.6		Unknown
Lifitegrast Na	1,295.3 ± 25.1	3	>5,000	3	>3.9		2.7 ± 2.1 nM in blood, 143 ± 67.5 μM in tears
Velpatasvir						500 μM: toxic, 100 μM: inactive	293 ± 61.2 nM
Cefpiramide	>1500	1	> 1000	2		800 μM: ∼50% active	88 μM
Simeprevir			16.4 ± 0.8	2		100 μM: toxic, 10 μM: inactive	2.59 μM
Lumacaftor	84 ± 3.7	3	314.5 ± 22.7	3	3.7		5.42 μM
Acalabrutinib	183.8	1	405.7 ± 21.2	2	2.2	100 μM: inactive	1.77 μM
Venetoclax			4.9 ± 2.2	2		10 μM: 40 % active	2.4 ± 1.3 μM
Ledipasvir			>1,000	2		400 μM: inactive	406 nM
Suramin	46.2 ± 1.6	3	>1,000	3	>21.6		1.89 μM

a Vero-E6 cells were incubated with virus and compound for 24 h (50% inhibitory concentration [IC_50_] and 50% cytotoxic concentration [CC_50_] data) to 48 h (screening data) at 37°C. Infection was measured with *in situ* ELISA, and IC_50_ values were determined by nonlinear regression of dose-response curves. The CC_50_ values were determined with an alamarBlue assay in identical experimental conditions.

b SI, selectivity index: SI = CC_50_/IC_50_.

c Indicative of published *C*_max_ values.

10.1128/mBio.03681-20.5 FIG S5 Dose-dependent inhibition of SARS-CoV-2 *in vitro* infection of Vero-E6 cells and cytotoxicity of approved drugs. Vero-E6 cells were incubated with virus and compound dilutions for 24 h at 37°C. Infection was measured with *in situ* ELISA using a primary SARS-CoV-2 nucleocapsid antibody and 50% inhibitory concentration (IC_50_) values determined by nonlinear regression of dose-response curves. The 50% cytotoxic concentration (CC_50_) values were determined with an AlamarBlue assay in identical experimental conditions. Data points represent the average ± SD of 1, 2, or 3 independent experiments each performed in technical triplicates, as indicated by the *n* values on the graphs. Download FIG S5, PDF file, 0.2 MB. Copyright © 2021 Day et al. 2021 Day et al. https://creativecommons.org/licenses/by/4.0/ This content is distributed under the terms of the Creative Commons Attribution 4.0 International license.

## DISCUSSION

The purpose of this study was to conduct a combined *in silico* and biophysical compound library screen for potential entry inhibitors that bind to the receptor for SARS-CoV-2, ACE2, and the SARS-CoV-2 S-spike proteins. *In silico* screening approaches have been taken by others for established targets, such as SARS-CoV-2 3C-like protease (3CL^pro^) (12), and also against the SARS-CoV-2 S-spike protein interaction domain on ACE2; however, no *in vitro* or *in vivo* tests have been reported (9, 20, 24). We are unaware of physical compound screens, which have targeted ACE2 for the identification of potential entry inhibitors that may function like the HIV-1 entry inhibitor maraviroc (1). Several *in silico* screening studies have been reported against SARS-CoV-2 spike protein (11) with hits including pralatrexate, carumonam, bradykinin, aclerastide, and granotapide and without *in vitro* or *in vivo* validations. A virtual screen of 640 antiviral compounds from the ChEMBL database against the trimeric S protein RBD-ACE2 complex (30) revealed two binding drugs (PC786 and zanamivir) binding at the interface of the trimer and no further *in vitro* or *in vivo* data.

As an adjunct to these studies, we also developed a biophysical assay to assess the potential for identified compounds to block the SARS-CoV-2 spike protein RBD-ACE2. Remarkably, the 17-amino acid peptide RBD-mimic1 recapitulated the binding affinity recently reported for the spike protein RBD-ACE2 complex (5). These data indicate that RBD-mimic1 is a functional, and presumably also a structural, mimic of the crucial, ACE2-interacting aspect of the RBD. This peptide may be a useful research reagent in serological studies and as an antigen in vaccine studies to generate neutralizing antibodies that block the SARS-CoV-2 RBD–ACE2 binding activity.

In this study, we report a series of ligands that bind with *K_D_*s in the low nM to low μM range to the human ACE2 protein. Molecular modeling supports the hypothesis that these compounds bind in the same region of ACE2 that SARS-CoV-2 uses as a cellular receptor. The predicted location of the binding of these compounds is confirmed by competition studies (>65% blocking) with RBD-mimic1 peptide, which demonstrates competition by 7 of the 11 compounds tested (Table 1). We also reported six compounds that interact with the SARS-CoV-2 spike protein. All six of these drugs show ∼70% blocking in the SPR ACE2-SARS-CoV-2 spike protein competition assay (Table 2). Of the 13 compounds that showed blocking by binding to either ACE2 or Spike RBD, 2 did not progress further, dactinomycin, a highly toxic chemotherapeutic for a wide range of cancers (31), and zotarolimus, a nonsoluble stent protective agent (32). The remaining 11 compounds were tested in Vero-E6 cell assays. The Vero-E6 model was selected for its well-established permissiveness to SARS-CoV-2 infection, as well as its high relative expression of cell-surface ACE2 that is crucial for SARS-CoV-2 infection (33, 34). Initial screening was performed at a concentration range of 1 to 1,000 μM to identify compounds that were nontoxic in the Vero-E6 assay and that showed activity warranting further experimentation. This screen produced two groups, seven drugs that showed cytotoxic activity in Vero-E6 cells that could not be further evaluated and four compounds where blocking activity could be tested (Table 3 and Fig. 4).

Over half of the compounds identified for testing in cell-based assays were toxic to the Vero-E6 cells. In some cases, the Vero cell toxicity is evident at concentrations well below the known human therapeutic *C_max_* (maximum concentration of drug in serum) of the same drugs in clinical use. Three of the Vero-E6-toxic compounds with blocking activity are registered antihepatitis C therapeutics, simeprevir, ledipasvir, and a related drug, velpatasvir (Table 1). Velpatasvir and ledipasvir were both recently identified as potential inhibitors of another SARS-CoV-2 target, the 3C-like protease (3CL^pro^) (12). Simeprevir is a registered hepatitis C drug that acts via inhibition of the viral protease. Simeprevir has also been identified as a SARS-CoV-2 inhibitor in several *in silico* screens against a range of viral proteins (7, 8, 15–20, 35–37). One of these *in silico* screens identified simeprevir as a binder to the same RBD region of the SARS-CoV-2 protein that we have also identified (20), and it was hypothesized that simeprevir may block the interaction with ACE2 in combination with lumacaftor (20). Irinotecan is a chemotherapeutic drug that showed complete blocking in our SPR competition assay but was also toxic to the Vero-E6 cells. Irinotecan is a prodrug that is metabolized *in vivo* to its active form, SN-38, which is a potent topoisomerase I inhibitor (38). Based on modeling (Fig. S1D), the SN-38 form is unlikely to have the same ACE2 binding activity once it has been processed from the prodrug form. No other screens, virtual, cellular, or biophysical, have identified irinotecan as a SARS-CoV-2 interacting compound. Bruton tyrosine kinase (BTK) inhibitors have been suggested and tested as a treatment for COVID-19, specifically for mitigation of the cytokine storm (39). Of the BTK inhibitors clinically tested to date, the most promising appears to be acalabrutinib (39). Our demonstration of the potential for viral entry blocking by acalabrutinib suggests that this activity may contribute to its apparent efficacy in treating COVID-19. Venetoclax is a B-cell lymphoma-2 (BclII) protein inhibitor that is effective against chronic lymphocytic leukemia (CLL), small lymphocytic lymphoma (SLL), and acute myeloid leukemia (AML) (40) and that has no previously identified interaction with SARS-CoV-2 interacting partners. In summary, our biophysical data indicate potential entry blocking activity with drugs that could not be tested in the Vero-E6 system, and these remain valid candidates that require further evaluation in other model systems of SARS-CoV-2 infection. Several screens for therapeutics for SARS-CoV-2 have utilized high-throughput screening in the Vero-E6 model of infection (22, 23). Based on our observations described above, it is likely that these screens may have missed inhibitors due to Vero-E6-specific toxicity rather than a lack of entry-blocking activity. During the preparation of this manuscript, Clausen et al. (41) reported that heparan sulfate polymers present on cellular proteoglycans can bind to spike protein of SARS-CoV-2, in addition to ACE2, to promote cell interaction. No heparin or heparin-related polymers were identified in our *in silico* or biophysical screens for spike RBD binders. Heparan sulfate proteoglycans are present on Vero-E6 cells that were used in the entry blocking assays presented here; therefore, any blocking activity that we report takes place in the context of cellular heparan sulfate.

The four compounds that were not Vero-E6-toxic were used in dose-response studies and were found to have IC_50_ values in the mid to high micromolar range. The initial biophysical and *in silico* screens that were conducted were restrictive to SARS-CoV-2 spike RBD/ACE2 blockade. Since only a few candidates were identified and were all approved drugs, they were screened for *in vitro* antiviral activity without using a cutoff concentration value to discriminate hits. Rather, all compounds demonstrating both anti-SARS-CoV-2 *in vitro* potency and low Vero-E6 toxicity were selected for more extensive dose-response experiments. There typically are stronger criteria for hit selection in high-throughput *in vitro* screens, such as low-micromolar cutoff or starting concentrations, since many more compounds are tested and a handful of compounds discriminated (42, 43). To the best of our knowledge, Evans blue, lifitegrast, and lumacaftor have not previously been identified through *in vitro* high-throughput screens, most likely due to cutoff concentration criteria that are higher than the IC_50_ reported in this article.

Cefpiramide is a broad-spectrum third-generation cephalosporin antibiotic that is delivered intravenously with a maximum dose of 2,000 mg that can achieve a *C_max_* of 205 μM from a single dose (44). Cefpiramide was found to bind to the SARS-CoV-2 spike protein with a *K_D_* of 330 nM with 81.9% blocking of the ACE2-RBD interaction in SPR (Table 2). In the Vero-E6 assays, it had a CC_50_ value greater than 1 mM, but an IC_50_ could not be accurately determined (Table 3).

The highest-affinity binding compounds from screening were Evans blue (see Fig. 2) and a related dye, Chicago sky blue, which had lower binding affinity and blocking potential (Table 1; Fig. S2). To our knowledge, Evans blue has not been identified previously in published virtual or biophysical screening with ACE2 or any of the SARS-CoV-2 proteins. Evans blue has a long history of use in human medicine. In the mid- to late 20th century, it was injected intravenously (i.v.) in procedures to measure cardiac function (45) and to measure plasma volume (46). It was also used to identify premature rupture of membranes by intraamniotic injection of Evans blue into pregnant women (47). The Evans blue test and modified Evans blue test were used up until the end of the 20th century, in which Evans blue was administered orally, four drops of a 1% solution, as a screening test for aspiration in tracheostomized patients (48). Evans blue is described as having a high affinity for human albumin, which has been reported to be in the low μM range (49). Here, we report a *K_D_* for human ACE2 that has an affinity that is 1,000-fold higher than for human albumin (*K_D_*, 1.6 nM; see Table 1). A recent review has detailed the potential for Evans blue in biomedical applications (50), including imaging in cancer (51, 52). Vero-E6 cell infection assays for SARS-CoV-2 show that Evans blue has an IC_50_ value of 28.1 μM, with a CC_50_ of greater than 1 mM. This was the most effective compound we tested, with a better IC_50_ than suramin, a previously reported inhibitor (29) that was used in our studies as a positive control.

The highest-affinity compound identified for binding to SARS-CoV-2 spike protein was lifitegrast (*K_D_*, 1.6 nM; Fig. 3; Table 2), a compound used to treat keratoconjunctivitis sicca and administered as eye drops. Two other groups using *in silico* screening identified lifitegrast as a compound that may bind to other SARS-CoV-2 targets, Nsp16 methyl transferase catalytic subunit (14) and the Nsp13 helicase (21). In the Vero-E6 cell assay, we showed that sodium lifitegrast had a 50% viral inhibition concentration of 1,295.3 μM. We note that sodium lifitegrast is currently used therapeutically as a 78-mM solution applied directly to a mucosal surface, i.e., 60-fold higher than the identified IC_50_.

Lumacaftor is a treatment for cystic fibrosis by aiding the conformational stability of the F508-_del_ mutated cystic fibrosis transmembrane conductance regulator (CFTR). As reported above, lumacaftor has been previously identified as an RBD spike protein binder (20). While lumacaftor had a lower affinity for the spike protein than many of the other compounds tested (1.51 μM; Table 2), it had an IC_50_ value of 84 μM in the Vero-E6 cell assays, the second-best inhibitor in the Vero-E6 assays of the compounds identified in our studies.

In summary, the compounds identified in this study are candidates for further evaluation in primary human airway cellular model systems and ACE2-humanized animal models as SARS-CoV-2 entry inhibitors. Given the limitations of the Vero-E6 model, which is nonrespiratory, more susceptible to drug-induced cytotoxicity, and lacking antiviral immune response, testing the identified compounds in more relevant model systems with a functioning immune response may generate synergies that improve IC_50_ compared with those observed for Vero-E6 cells. The compounds identified here include high-affinity ligands of ACE2 and spike protein that are registered drugs, and a dye used in biomedical applications, that may be candidates for repurposing or as chemical scaffolds for drug development to generate entry blockers to prevent or cure COVID-19.

## MATERIALS AND METHODS

### Molecular docking screening.

**Accession of target protein and box selection.** The three-dimensional structure of human ACE2 was obtained from the protein databank (http://rcsb.org) using the structure of SARS coronavirus spike RBD complexed with the human ACE2 receptor (PDB 2AJF) (53) at 2.9 Å resolution. A rectangular box with dimensions 50 Å by 60 Å by 40 Å (*x*, *y*, and *z*) was centered on the coordinate amino acids HIS-34 as seen in Fig. 1B. The human ACE2 structure was cleaned by deleting the SARS coronavirus spike protein and all water molecules. Subsequent to our screen, the region of SARS-CoV-2–ACE2 interaction was modeled (53), and it is consistent with a recent cryo-electron microscopy (cryo-EM) study of the SARS-CoV-2–ACE2 complex at 3.6 Å (5), showing that SARS-CoV-2 and SARS-CoV interact with a similar region of ACE2, i.e., the region boxed in Fig. 1.

The three-dimensional X-ray crystal structure of the SARS-CoV-2 chimeric receptor-binding domain complexed with its receptor human ACE2 was obtained from the protein databank (http://rcsb.org) (PDB 6VW1) at 2.68 Å resolution (28). A rectangular box with dimensions 50 Å by 60 Å by 50 Å (*x*, *y*, and *z*) was centered at the amino acid SER-494 of the SARS-CoV-2 S-spike protein coordinates (see Fig. 1C). The SARS-CoV-2 chimeric receptor-binding domain structure was cleaned by deleting the human ACE2 protein and all water molecules.

**Ligand selection.** Chemical structures of ligands were downloaded from multiple libraries (Approved Drugs, 4,195; Charitee Super Drugs, 1,050; eDrugs, 1,610; Ligandbox Kegg, 5,814; Prestwick Off-Patents, 2,062; Otava, 9,765; ChemDiv, 33,145) as a two-dimensional (2D) SDF (structure-data file) molecular format. Three-dimensional (3D) conformers of all ligands are needed for *in silico* screening and were generated using DataWarrior (version 4.7.2) software utilizing the MMFF94s+ forcefield (54). A total of 57,641 compounds were docked against human ACE2 with a total computing time of ∼7 days using the Griffith University high-performance computing cluster and two Windows workstations.

**Target and ligand optimization.** Molecular screening of the molecular database was performed using Autodock Vina (55) implemented in the YASARA software suite (56). The macro dock_runscreening.mcr was used and modified to dock the molecular library to human ACE2 (PDB 2AJF) and SARS-CoV-2 chimeric receptor-binding domain (PDB 6VW1) using 12 docking runs per ligand in a completely flexible mode with an average time requirement of 12 s per ligand using the Griffith University high performance cluster.

### Surface plasmon resonance (SPR).

SPR screening was performed as previously described (25). Briefly, SPR analyses of compounds binding to immobilized human ACE2 were done using a Biacore S200 system (GE Healthcare Life Sciences). Recombinant, human ACE2 sourced from two separate companies (Assay Matrix, R&D Systems) was immobilized onto separate cells of a Series S CM5 sensor chip, separately or as a mixture, using NHS (N-hydroxysuccinimide) capture within the amine capture wizard (GE Healthcare Life Sciences) at pH 4.0 with a flow rate of 5 μl/min and an immobilization time of 600 s at 25°C. Two libraries (Microsource-CPOZ, 2,400 compounds; ML Drug, 741 compounds) comprising drugs, dyes, and other therapeutic molecules were purchased from Compounds Australia. Postscreen, kinetic analysis was performed to determine the affinity of binding (equilibrium dissociation constant, K_D_). Competition assays (ABA; injection method according to the manufacturer’s instructions; GE S200) were performed between the identified compounds in competition with a 17-amino acid peptide, RBD-peptide1, designed based on the model of SARS-CoV-2 spike protein (53) in complex with ACE2. The peptide comprises key interacting residues equivalent to the spike protein RBD (see Fig. 2A). The peptides RBD-mimic1 (*H-*NCYFPLQSYGFQPTNGV*-OH*) and RBD-mimic2 (*H-*NCYFPLQSYGFQPTNGVGY*-OH*) were custom synthesized by Mimotopes, Australia.

For the SARS-CoV-2 spike protein SPR, analyses were performed as outlined above except for the following. Empty VLPs were loaded onto flow cell 1 or 3 of a Series S CM5 sensor chip using an NHS capture kit (GE Healthcare Life Sciences) as the negative control for subtraction from the active flow cells 2 and 4. SARS CoV2 Spike protein-expressing VLPs were immobilized onto flow cells 2 and 4. Immobilization was performed at 5 μl/min for 12 min at pH 5.5. VLPs were captured at between 692 and 1,021 response units.

### Virus-like particles (VLPs).

Noninfectious lentivirus-like-particles (VLPs) were produced in the presence or absence of SARS-CoV-2 spike protein to mimic the presentation of SARS-CoV-2 spike proteins on the surface of an enveloped virus. SARS-CoV-2 spike protein mammalian cell expression vector is a kind gift from Linda Wang at Duke-NUS. SARS-CoV-2 spike protein coding sequences are codon-modified and based on the first published SARS-CoV-2 genome in GISAID (Accession ID EPI_ISL_402119). Lentivirus-like particle expression vector was modified from full-length HIV proviral plasmid DNA by deleting the coding sequencing of HIV reverse transcriptase, integrase, and Vif and Vpr genes. A termination codon was introduced at the end of the protease coding sequence via PCR mutagenesis. The restriction sites ApaI and EcoRI were used for this part of the cloning procedure. The initiation codon of Vpu has also been changed from ATG to CTG using PCR mutagenesis, thereby blocking Vpu expression. An early termination codon has been introduced into the C4 segment of the surface (gp120) segment of HIV Env at the StuI restriction site to prevent expression of functional of HIV Env. The resulting plasmid has been denoted NL ΔRTΔIN Env(-) to highlight the major deletion/inactivation. Transfection of NL ΔRTΔIN Env(-) into mammalian cells will lead to production of noninfectious HIV particles that lack 6 out of 15 viral genes, while cotransfection of NL ΔRTΔIN Env(-) with the SARS-CoV-2 spike protein expression vector in mammalian cells will generate SARS-CoV-2 spike protein pseudotyped VLPs. Production of VLPs and SARS-CoV-2 spike pseudotyped VLPs was done by polyethyleneimine (PEI)-mediated transfection of plasmid DNA into HEK 293 cells, and purification of these VLPs was done using the virus purification procedure that we have previously described for HIV (57, 58).

### Cells and virus.

Vero-E6 cells were maintained in advanced minimal essential medium (MEM) supplemented with 5% fetal bovine serum (FBS) at 37°C in a humidified atmosphere of 5% CO_2_. SARS-CoV-2 strain SARS-CoV-2-CoV-2/Australia/QLD02/2020 (GISAID accession code EPI_ISL_407896) was obtained from the Forensic and Scientific Services Unit of Queensland Health, Australia. The virus was propagated in Vero-E6 cells in medium supplemented with 2% FBS (infection medium). All work involving live SARS-CoV-2 cultures was carried out in a certified physical containment level 3 (PC3) facility at the Institute for Glycomics, Griffith University.

### Virus propagation and titration.

SARS-CoV-2 stocks were prepared by infecting confluent Vero-E6 cells at a multiplicity of infection (MOI) of 0.05 for 72 h at 37°C. Infection supernatants were clarified by centrifugation at 4,000 × *g* for 15 min, homogenized, aliquoted, and stored at −80°C. Virus stock titers were determined by focus-forming assays as follows: confluent Vero-E6 cells in 96-well plates were infected with 10-fold dilutions of virus in 50 μl for 1 h at 37°C, after which 50 μl of infection medium containing 1% Avicel (FMC BioPolymer) was added to each well. Plates were further incubated for 24 h at 37°C. SARS-CoV-2 foci were obtained by following the *in situ* enzyme-linked immunosorbent assay (ELISA) procedure detailed below, but by adding 50 μl per well of TrueBlue peroxidase substrate (KPL) in place of tetramethylbenzidine (TMB) reagent until dark blue foci appeared. Wells were subsequently rinsed with running water, and foci were manually counted to determine the focus-forming units (FFU) per ml.

### Biological screening of drug candidates.

Drug screening was done following similar methods previously described for SARS-CoV-2 (59) and another virus (60). Briefly, Vero-E6 cells were seeded in 96-well plates at a density of 1.75 × 10^4^ cells per well. On the day of infection, the medium in each well was removed and replaced with the subsequent addition of 50 μl of infection medium, 25 μl of compound dilution in infection medium (30 min before infection), and 25 μl of SARS-CoV-2 dilution. The final volume in each well was 100 μl, and infection was done at an MOI of 0.002. Virus and compound mixtures were left in place, and cells were incubated for 48 h at 37°C and 5% CO_2_ before infection was measured using *in situ* ELISA. Compounds were evaluated in technical duplicates.

### Dose-response experiments.

Assays were conducted as for the drug screenings, but Vero-E6 cells were infected at an MOI of 0.12 and infections carried out for 24 h to mitigate the risks of compound-induced cytotoxicity observed during longer incubation times. This MOI value was selected as it yielded a maximum signal falling within the upper linear range of an MOI-response curve measured with *in situ* ELISA. Infection in the presence of compound was measured with *in situ* ELISA, and compounds were evaluated in technical triplicates. The compound concentrations that inhibit 50% of SARS-CoV-2 infection (IC_50_ values) were determined by nonlinear regression of dose-response curves using GraphPad Prism 8.

### *In situ* ELISA.

ELISAs were adapted from previously published methods (60). Infected cells in 96-well plates were fixed by addition of 100 μl per well of an 8% paraformaldehyde solution in phosphate-buffered saline (PBS) for 30 min at room temperature. Cells were subsequently permeabilized and endogenous peroxidases inhibited with 1% IGEPAL and 0.3% H_2_O_2_ in PBS, respectively, for 20 min at 37°C. The intracellular SARS-CoV-2 nucleocapsid was immunostained by incubating cells with a 1:2,000 dilution of primary mouse anti-SARS-CoV-2 nucleocapsid antibody (reference no. [ref.] 40143-MM08; SinoBiological) in PBS/5% skim-milk for 30 min at 37°C, and a 1:6,000 dilution of secondary goat anti-mouse IgG(H+L)-horseradish peroxidase (HRP)-conjugated antibody (ref. 170-6516; Bio-Rad) in PBS/5% skim-milk for 30 min at 37°C. The cell monolayers were washed three times for 5 min with PBS/0.02% Tween 20 after each of the aforementioned incubations. Nucleoprotein levels were detected using 50 μl per well of OptEIA TMB substrate (BD Biosciences), and the reactions stopped with 25 μl per well of 0.6 M H_2_SO_4_. The absorbance at 450 nm was read in each well using an X-Mark microplate absorbance spectrophotometer (Bio-Rad). Percentages of infection were calculated by subtracting the background absorbance of negative-control wells (noninfected cells) from all other wells and normalizing the resulting values to positive-control wells (infected cells, not treated).

### Drug cytotoxicity assays.

Compound dilutions were incubated with Vero-E6 cells in 96-well plates in the absence of virus, in infection medium, for 24 h at 37°C. They were subsequently discarded, and the cell monolayers were washed twice with 100 μl of infection medium before applying 50 μl per well of 10% alamarBlue (Thermo Fisher) in serum-free advanced MEM. Plates were further incubated for 2 to 4 h at 37°C, and absorbances were read in each well at 570 nm and 600 nm using an X-Mark microplate absorbance spectrophotometer (Bio-Rad). Cellular viability was calculated following the manufacturer’s instructions and expressed as the percentage of control (untreated cells). The compound concentrations inducing 50% cytotoxicity (CC_50_ values) were determined by nonlinear regression of dose-response curves using the software GraphPad Prism 8. Compounds were evaluated in technical triplicates.
