# Supplementary material for: Multidisciplinary Approaches Identify Compounds that Bind to Human ACE2 or SARS-CoV-2 Spike Protein as Candidates to Block SARS-CoV-2–ACE2 Receptor Interactions
Source: mBio. 2021 Mar 30;12(2):e03681-20. doi: 10.1128/mBio.03681-20 (PMC8092326; doi:10.1128/mBio.03681-20)
Supplement: FIG S1 [file mBio.03681-20-sf001.pdf]

Figure S1 Molecular docking of the compounds identified for ACE2.
A. Ledipasvir

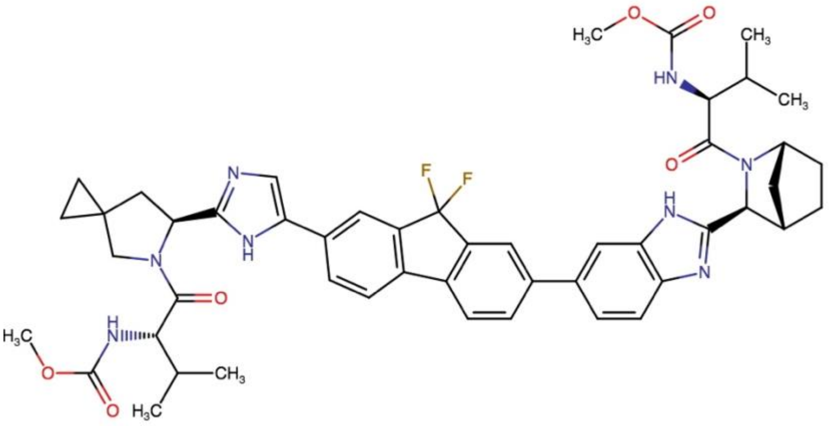

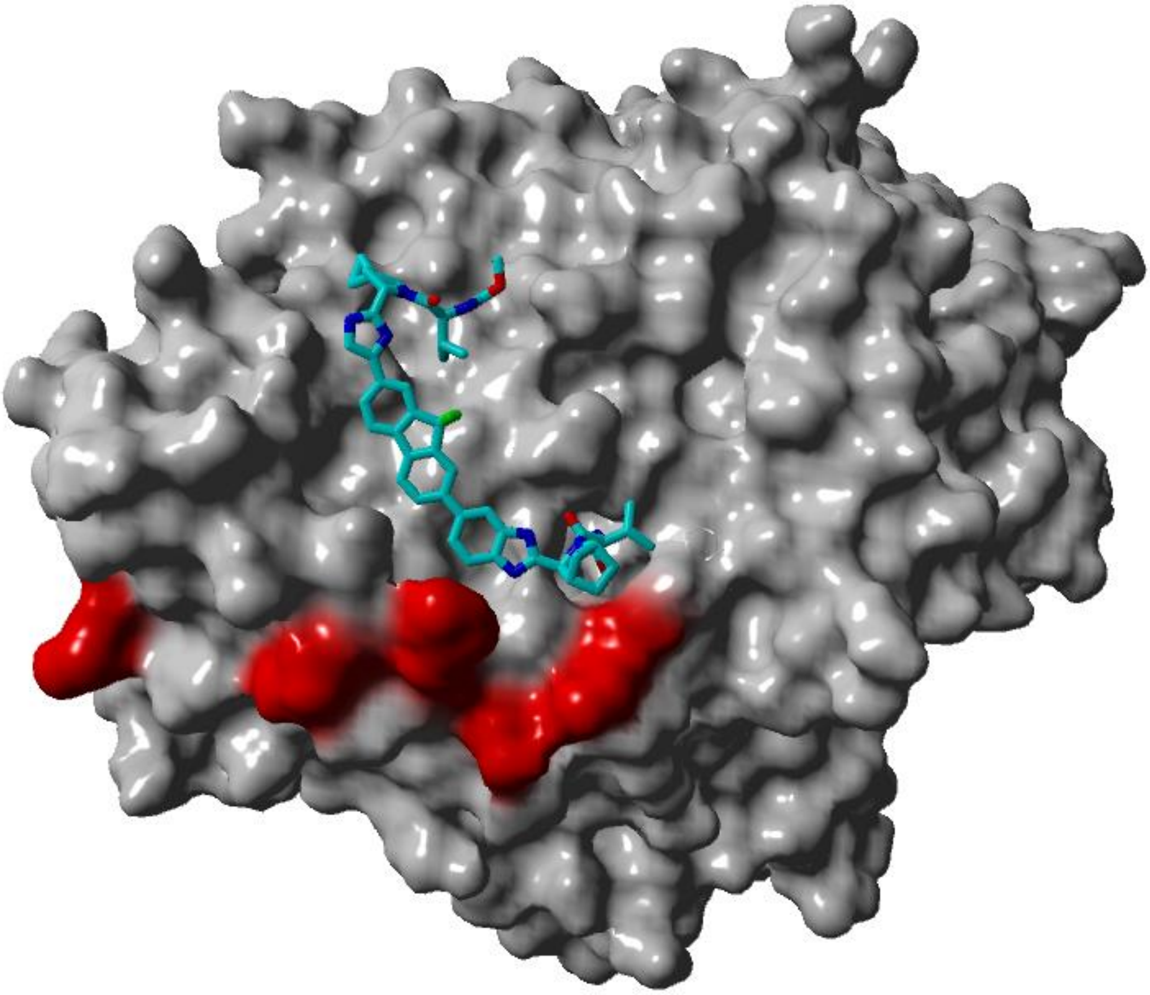

Predicted K$_D$: 232 nM

B. Venetoclax

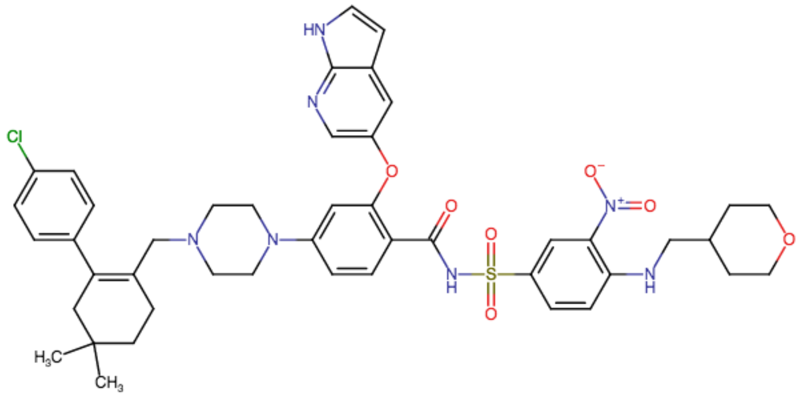

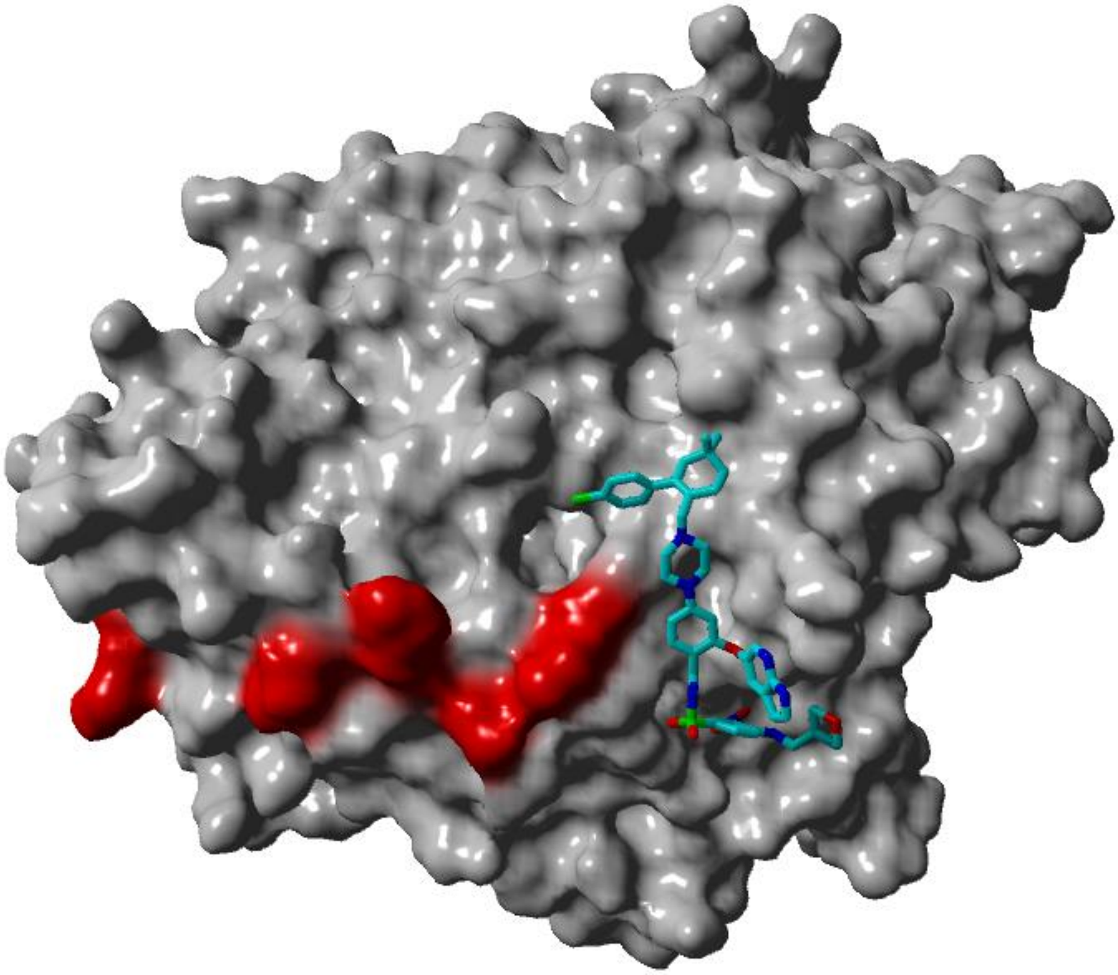

Predicted K_D: 412 nM

C. CID 3110549

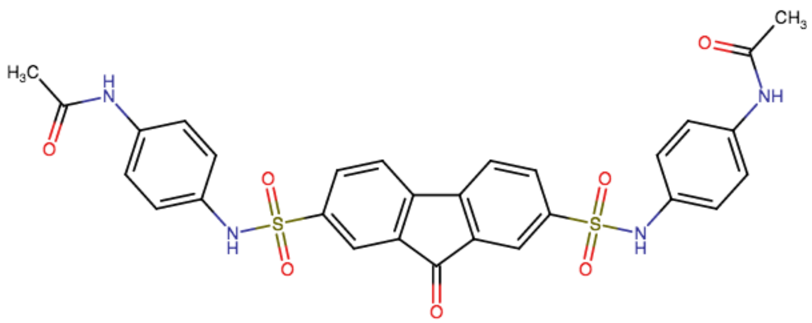

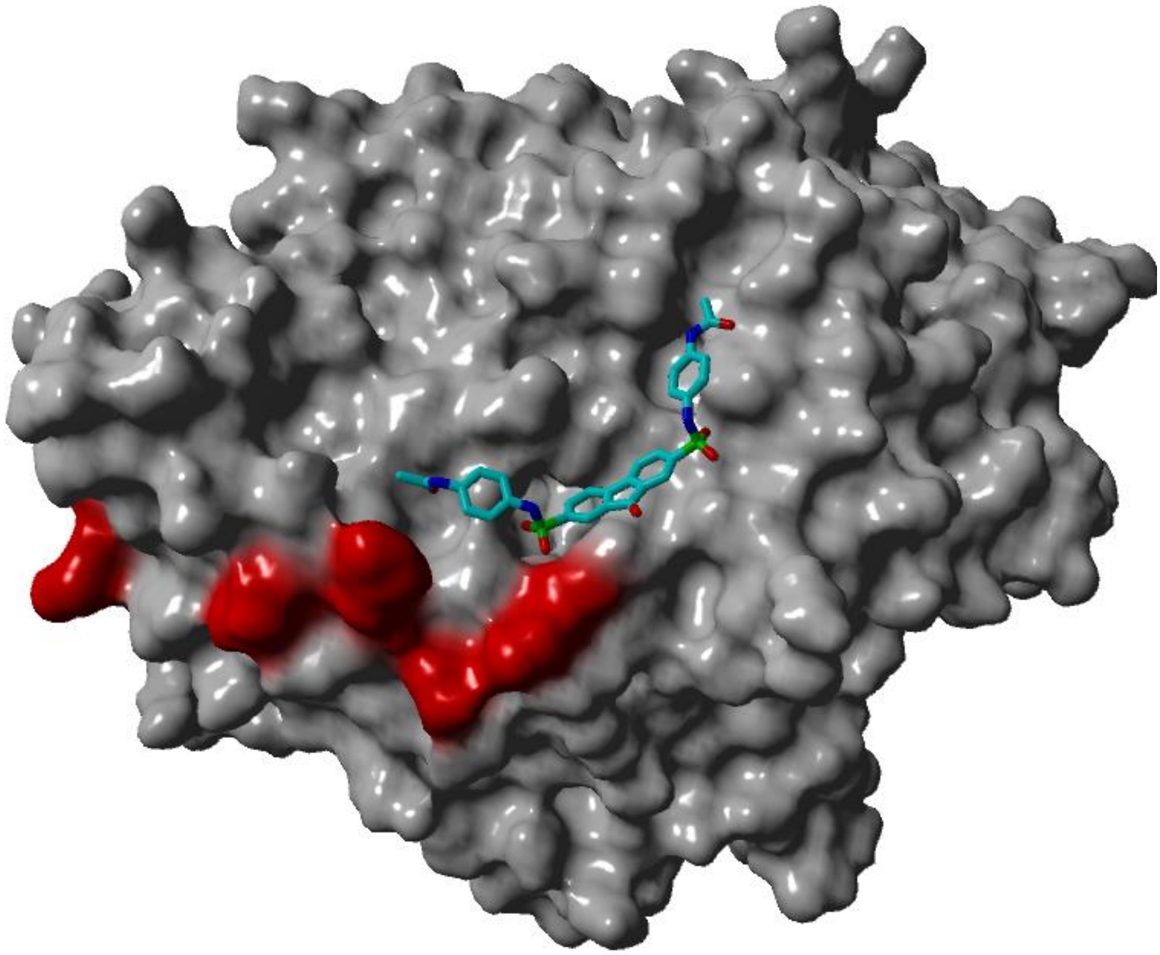

Predicted K_D: 557 nM

D. Irinotecan

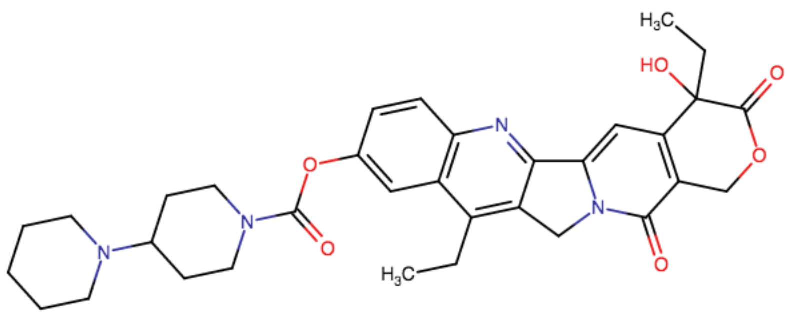

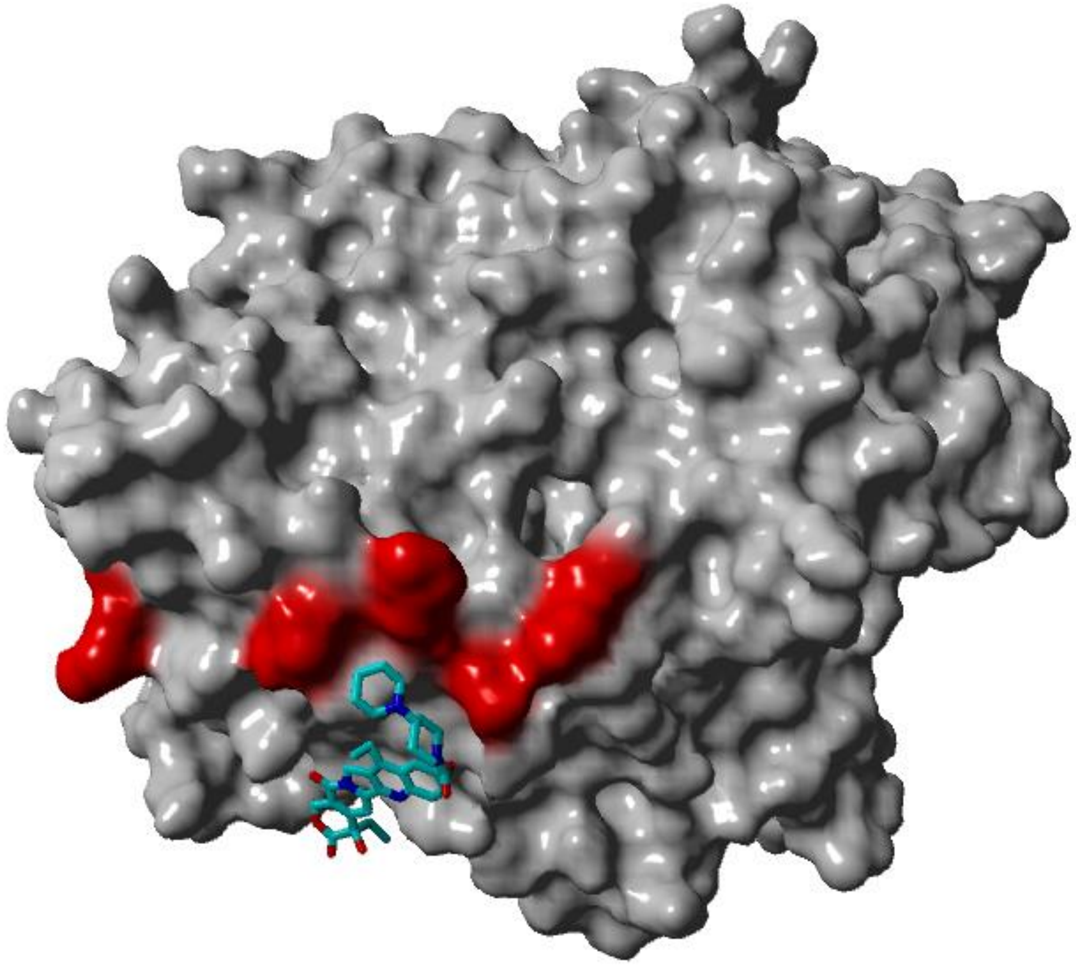

Predicted K_D: 577 nM

E. Digitoxin

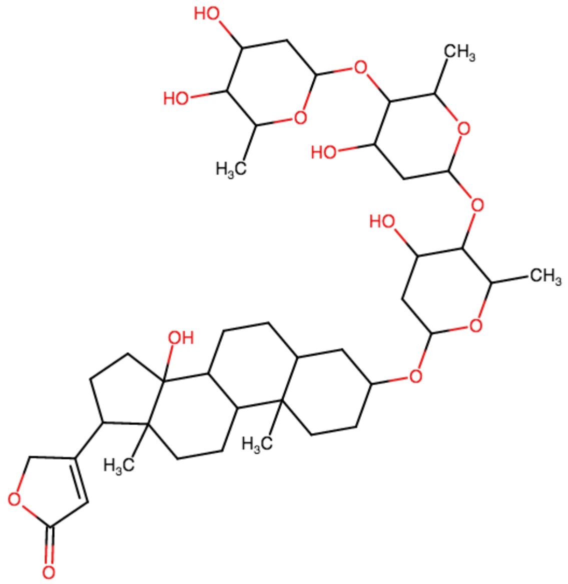

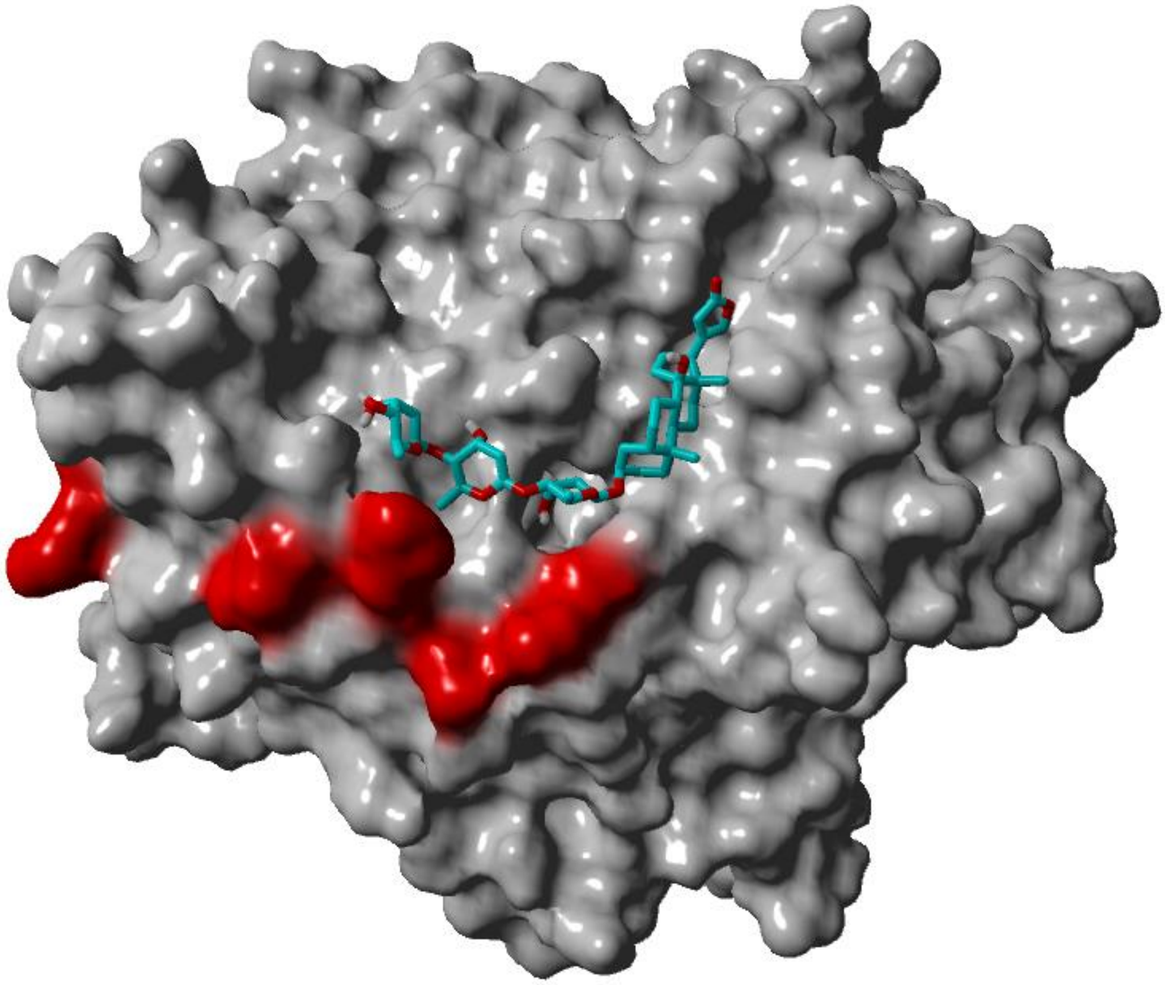

Predicted K_D: 581 nM

F. CID 16455811

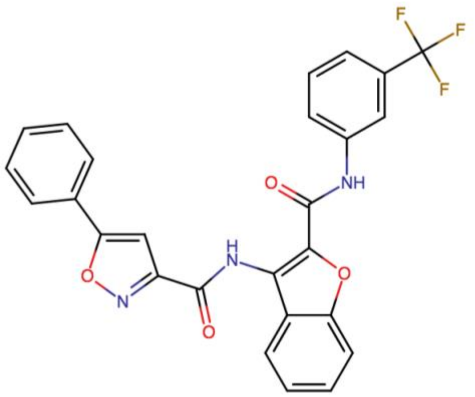

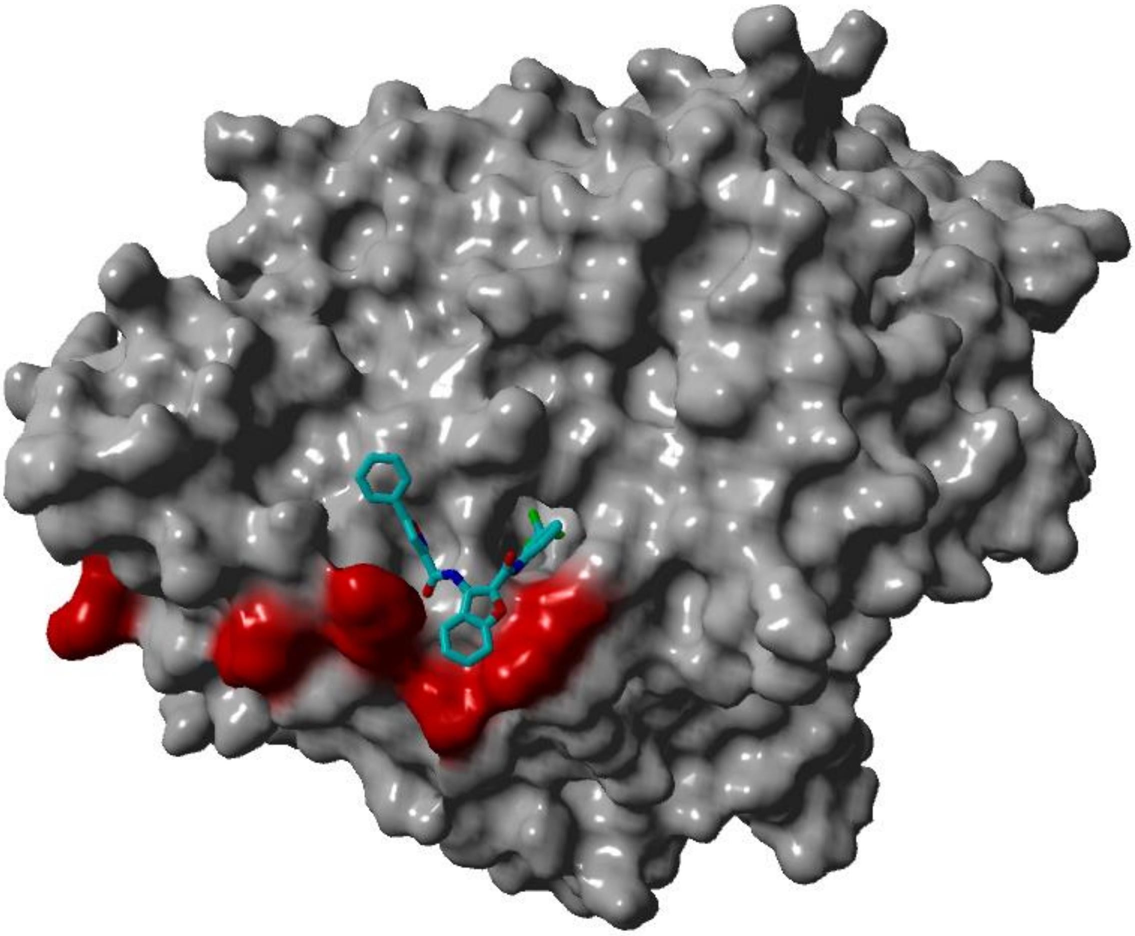

Predicted K_D: 859 nM

G. Gedatolisib

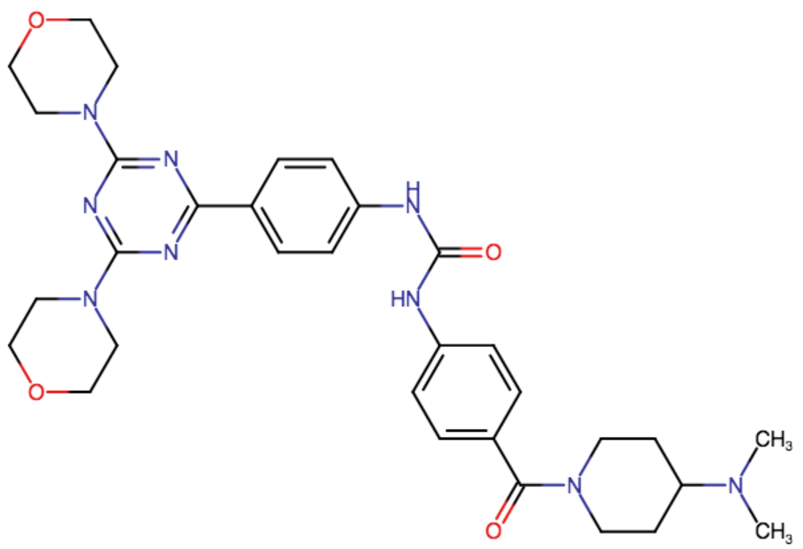

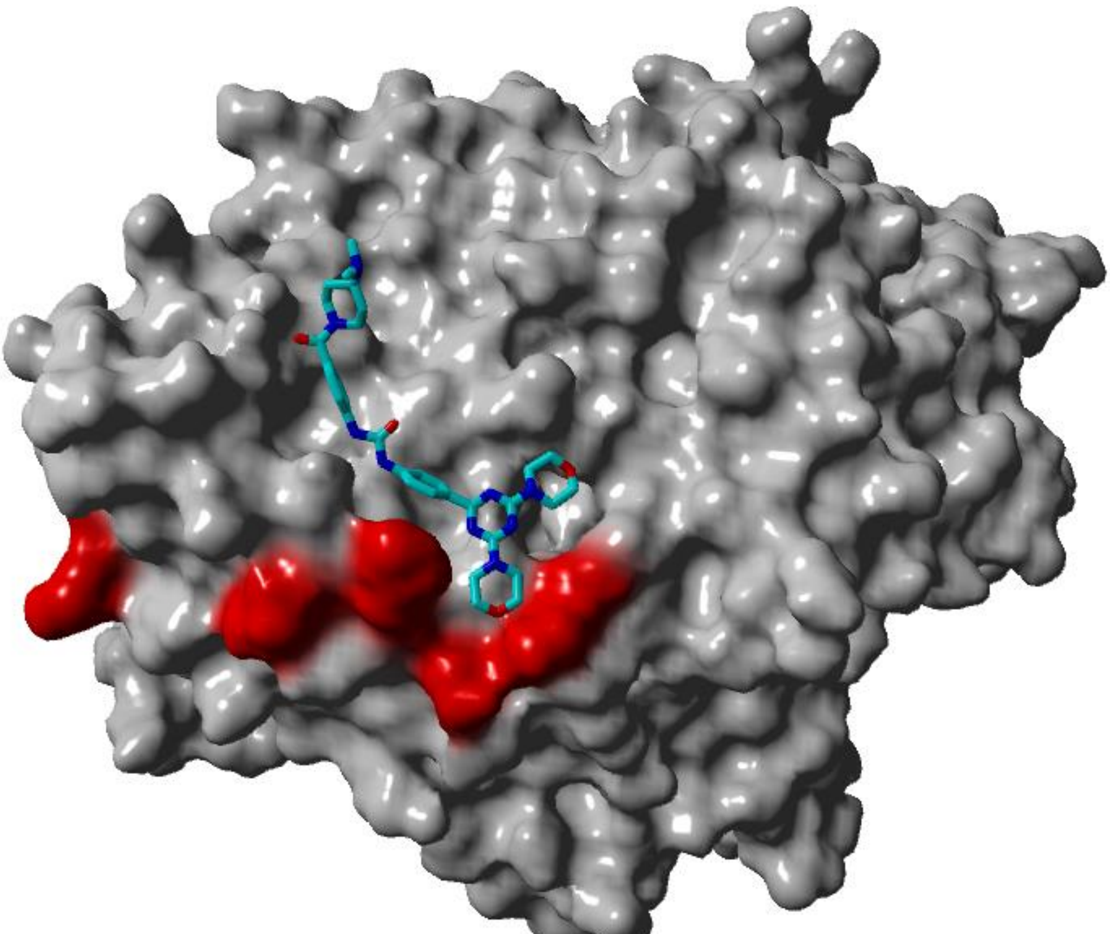

Predicted K_D: 1054 nM

H. Digoxin

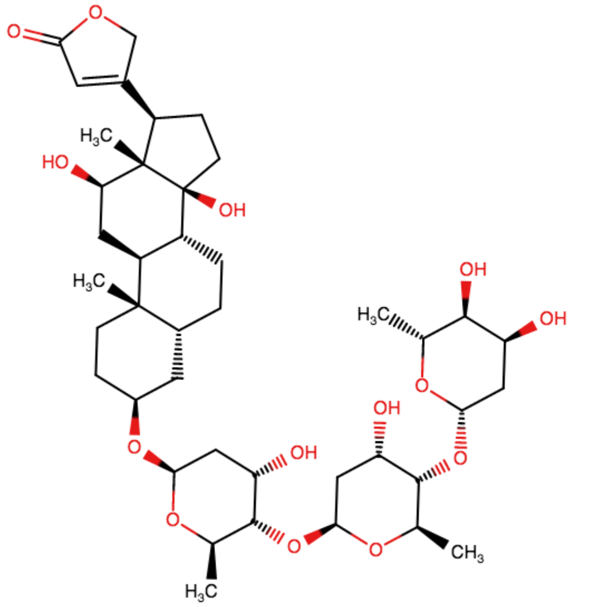

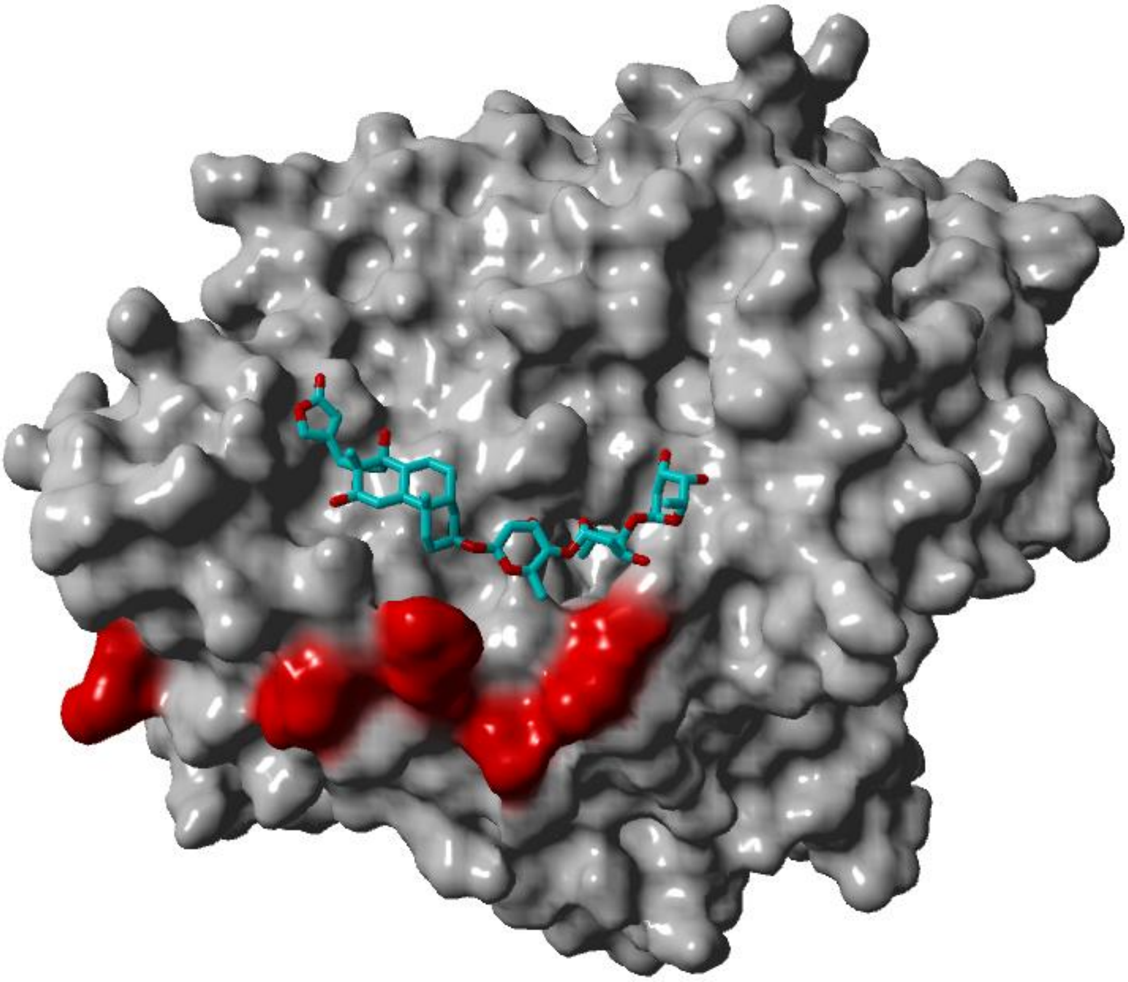

Predicted K$_D$: 1083 nM

I. Velpatasvir

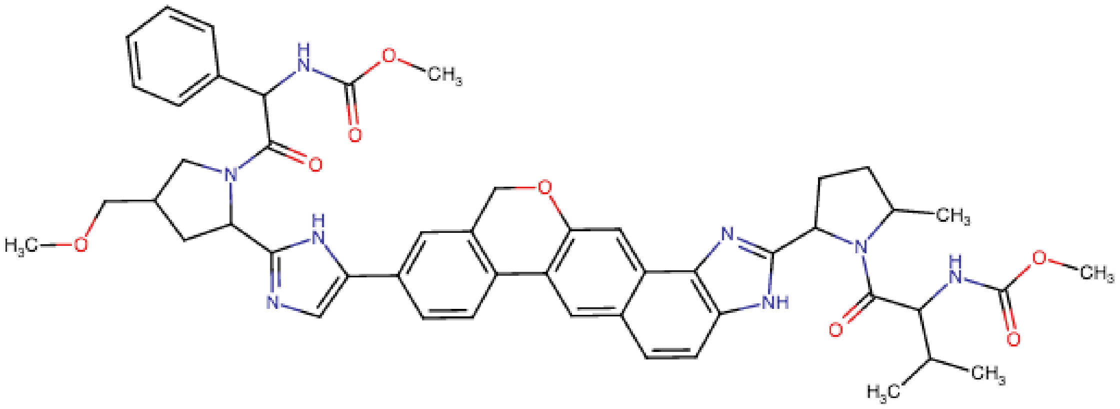

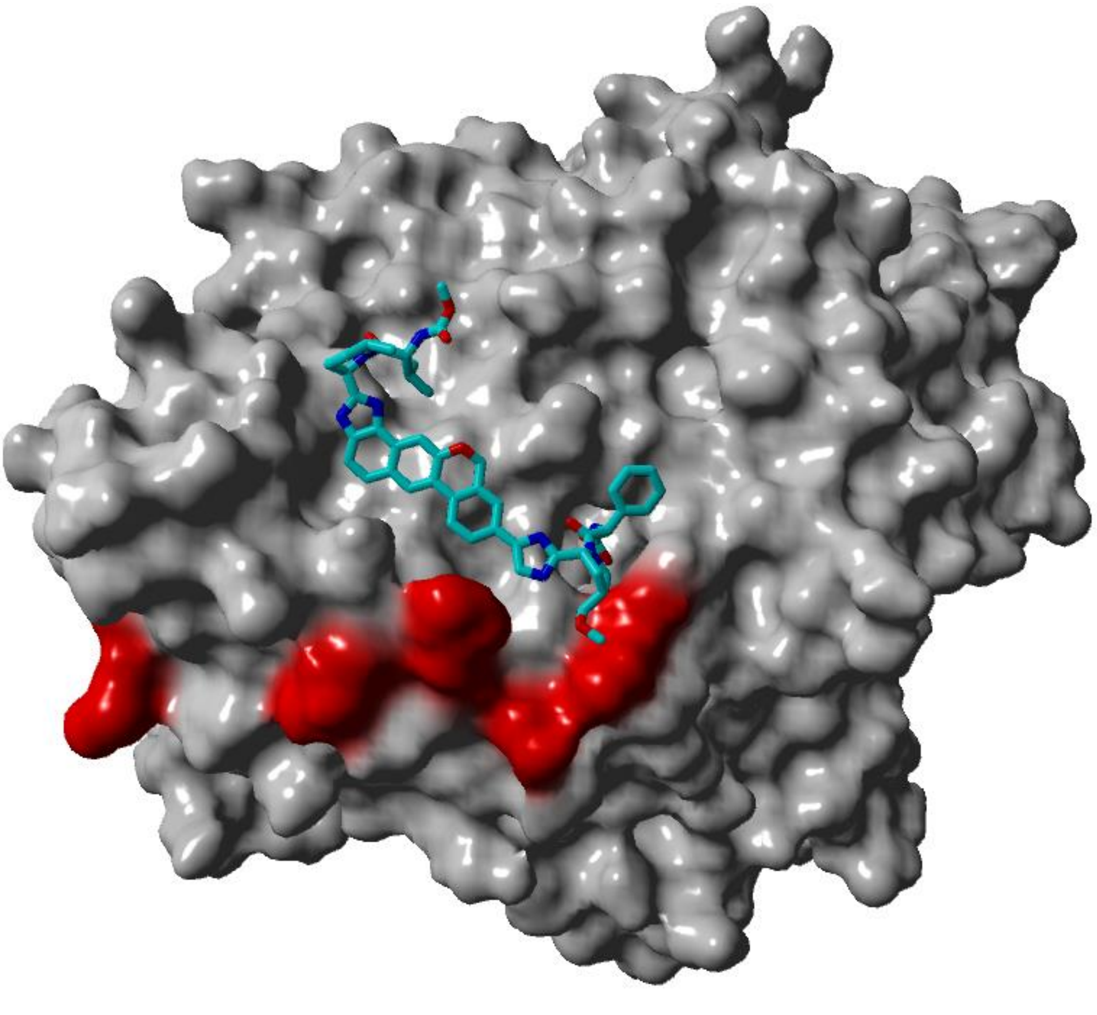

Predicted K_D: 1163nM

J. Radotinib

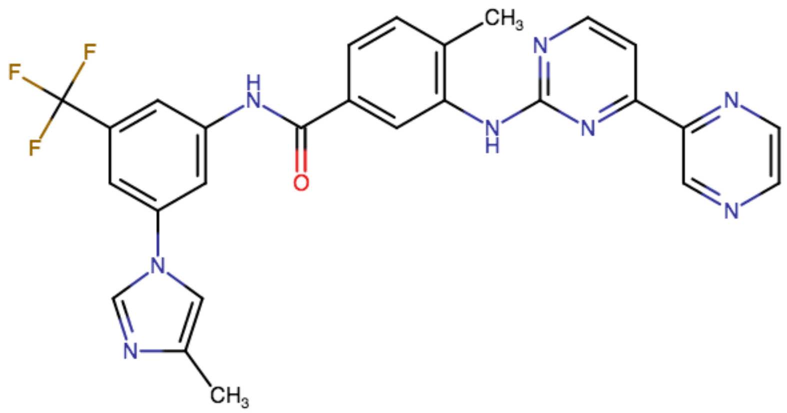

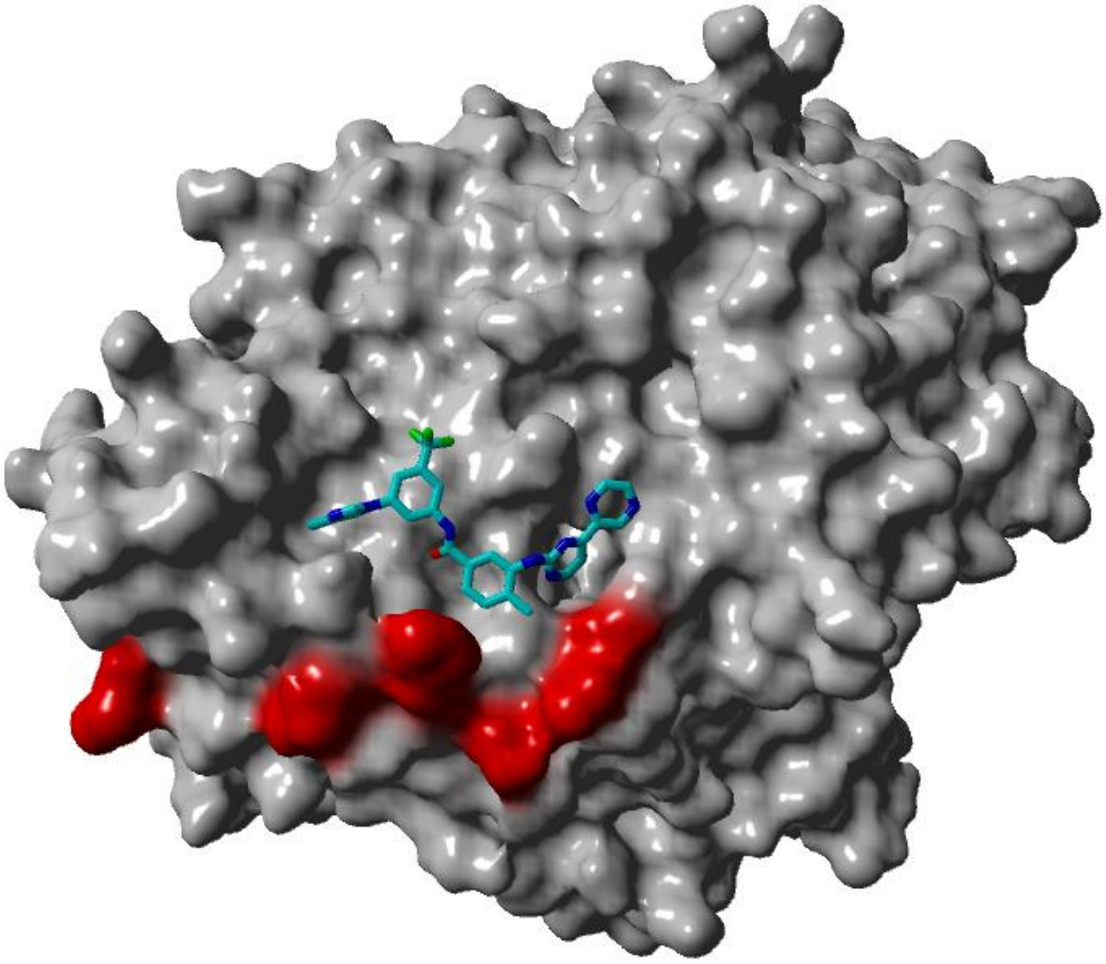

Predicted K_D: 1167 nM

K. Zotarolimus

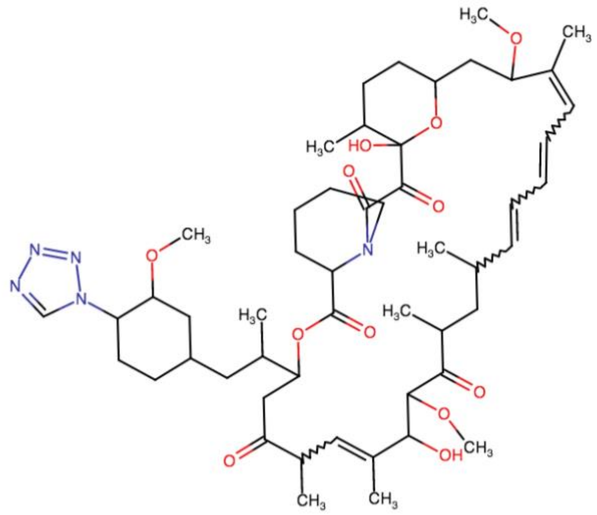

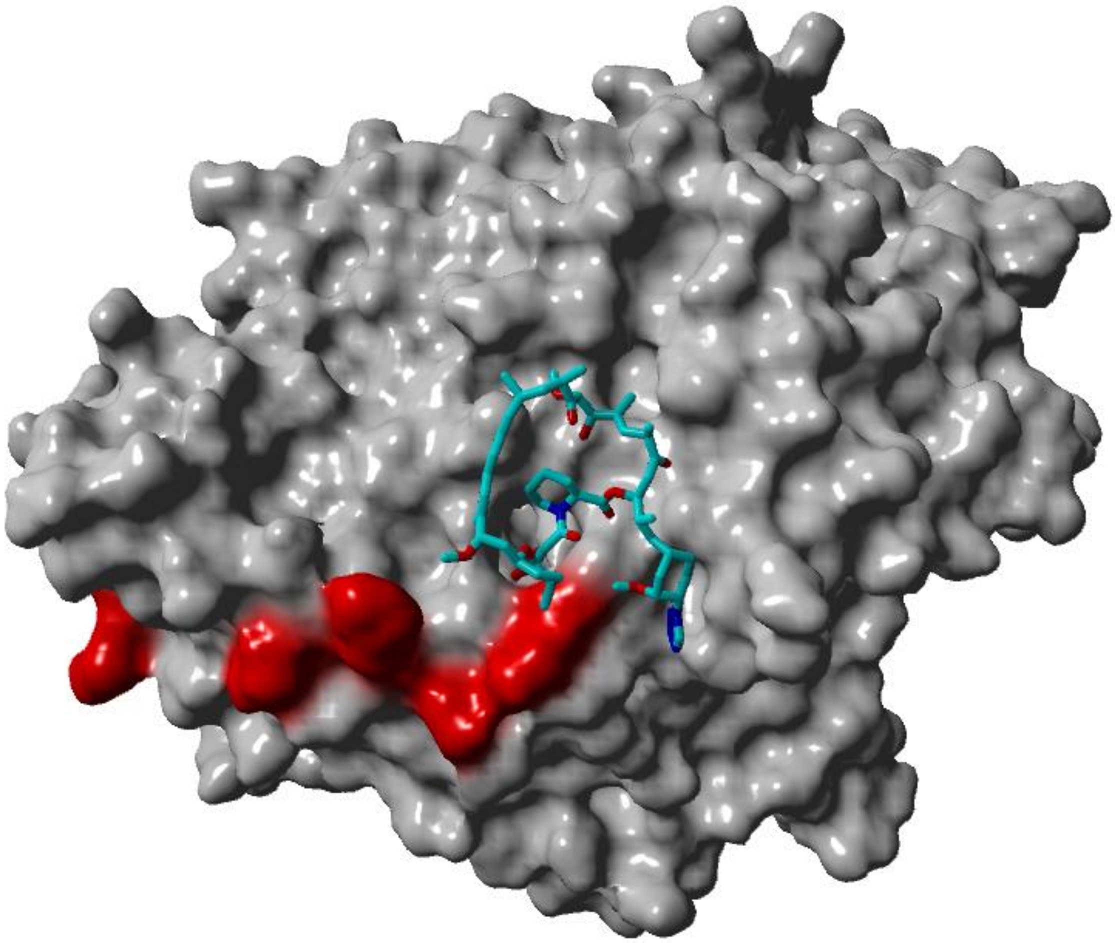

Predicted K_D: 1246 nM

L. Caspofungin

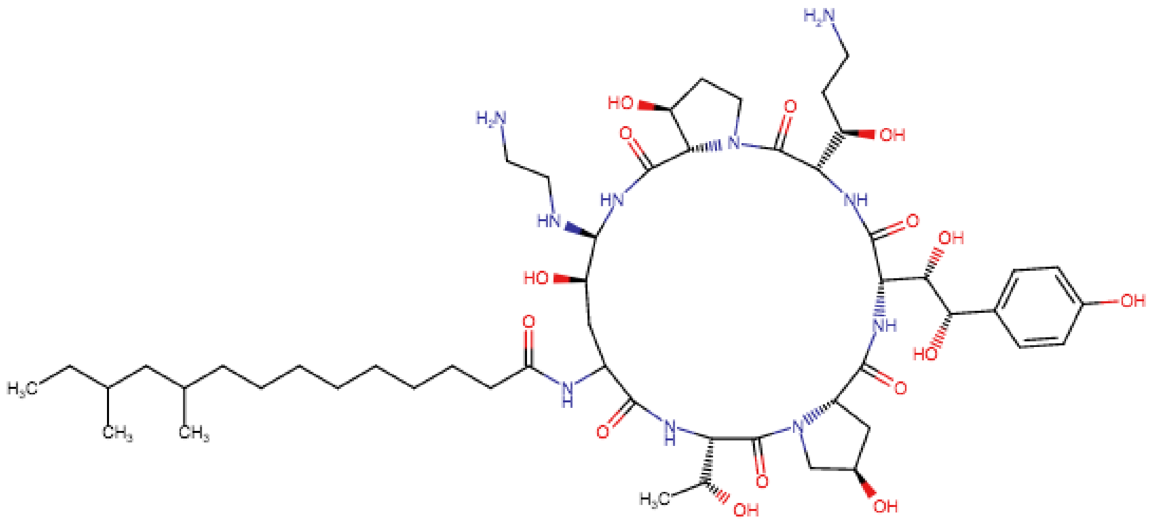

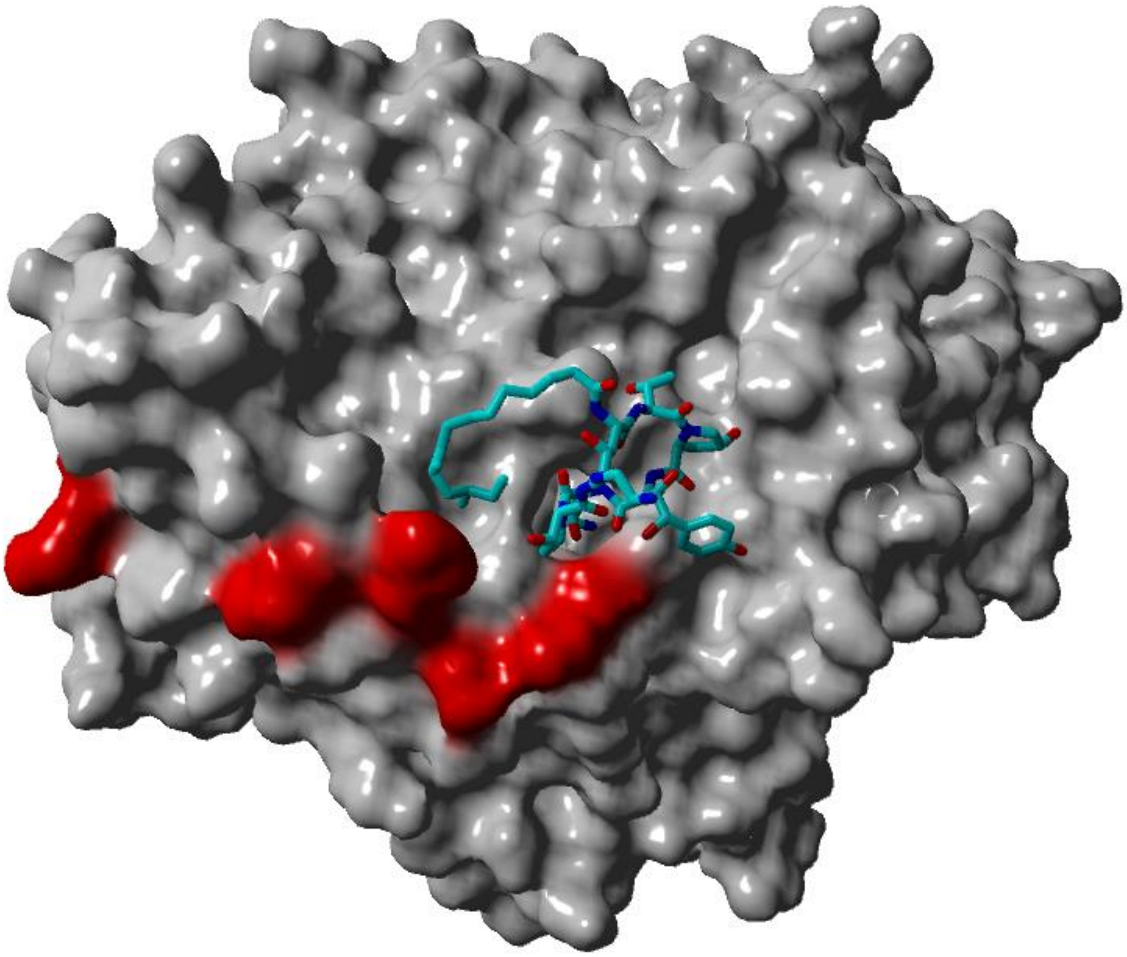

Predicted K_D: 3833 nM

M. Acalabrutinib

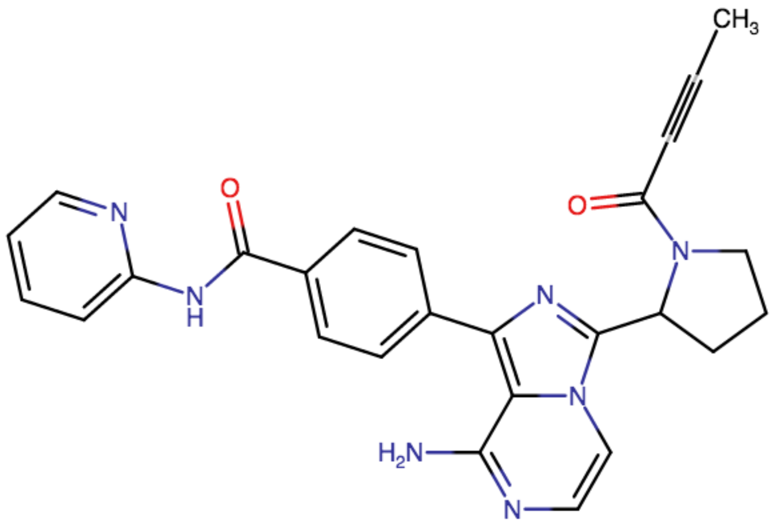

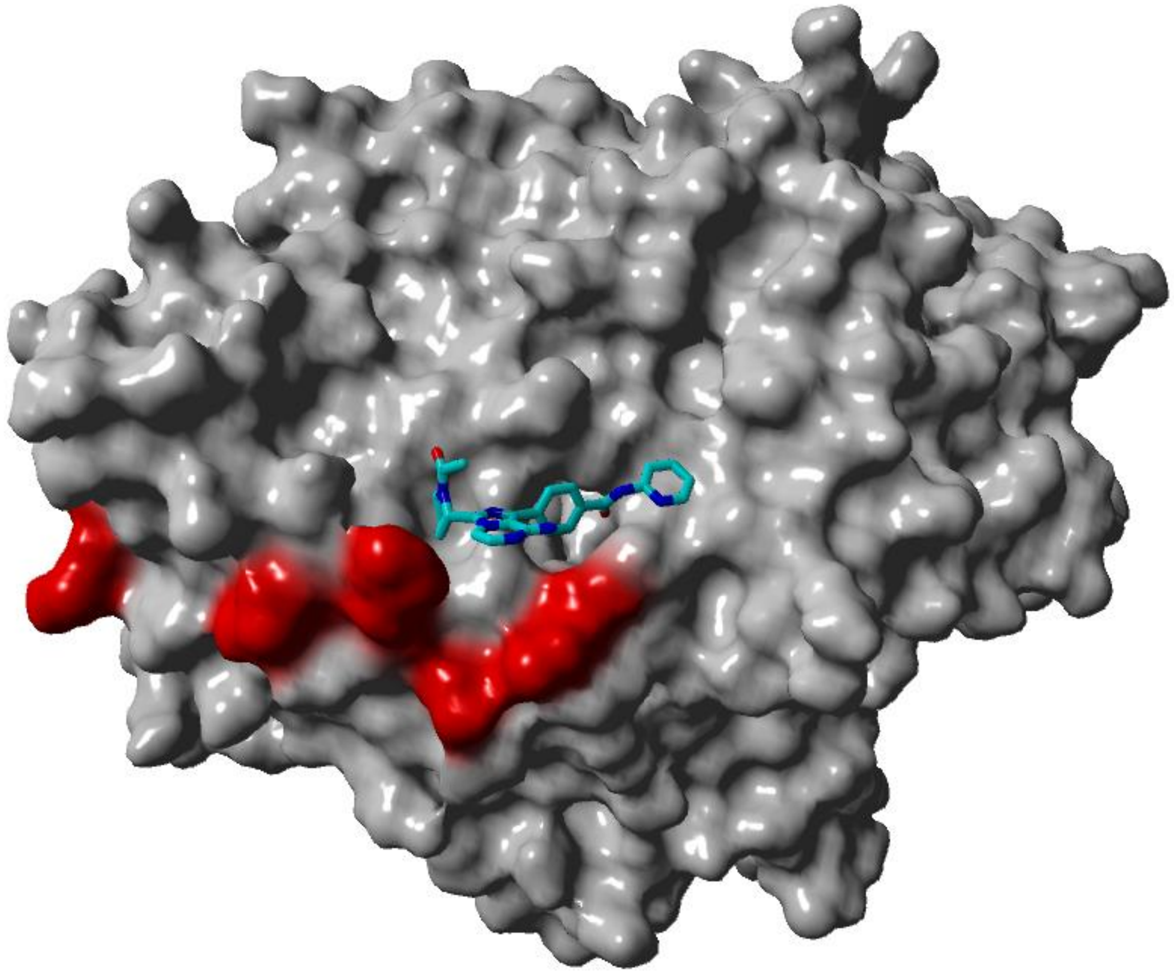

Predicted K_D: 5083 nM

N. Epigallocatechin-3-gallate

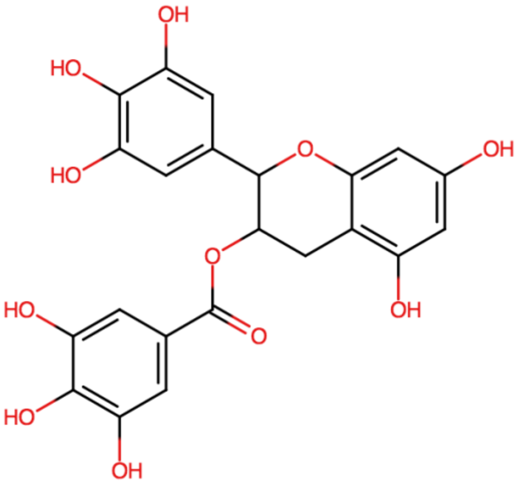

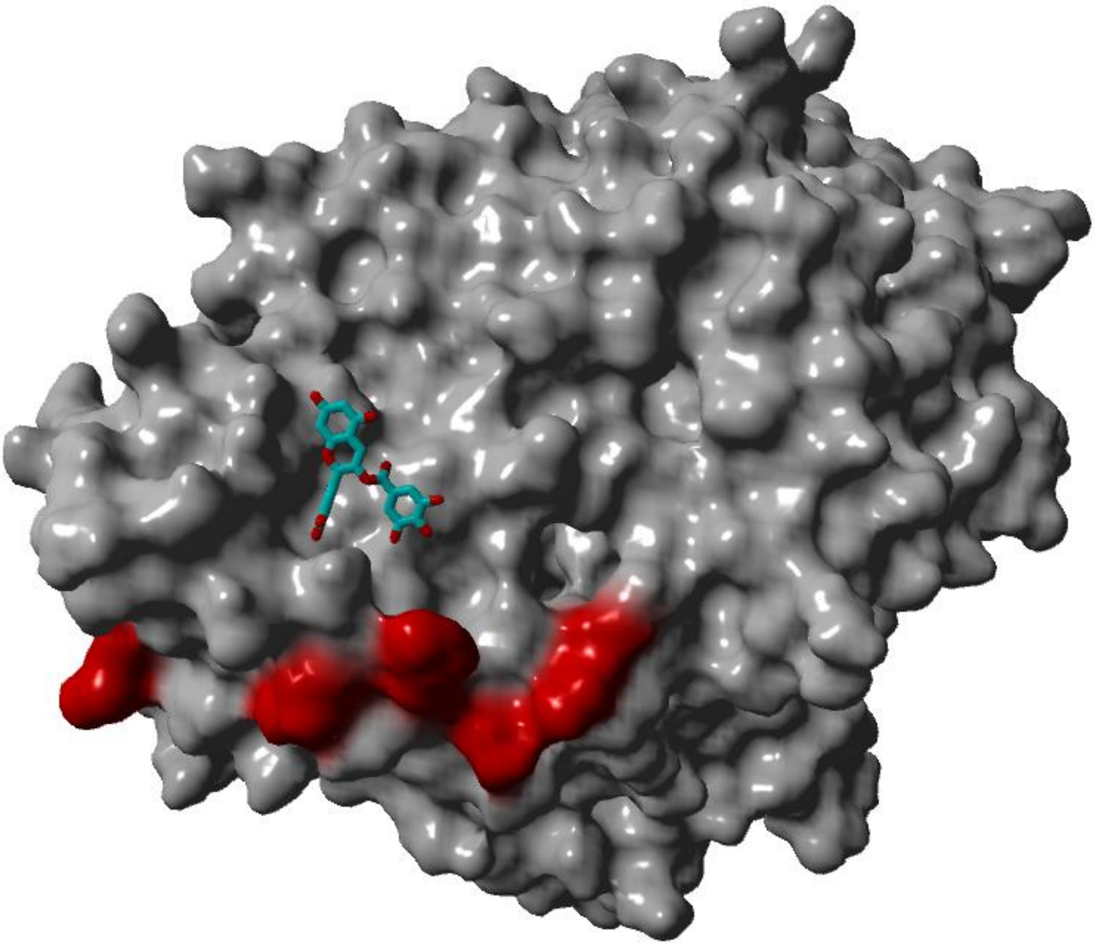

Predicted K_D: 3600nM

O. Evans Blue Mimetic (CAS-303106-55-0)

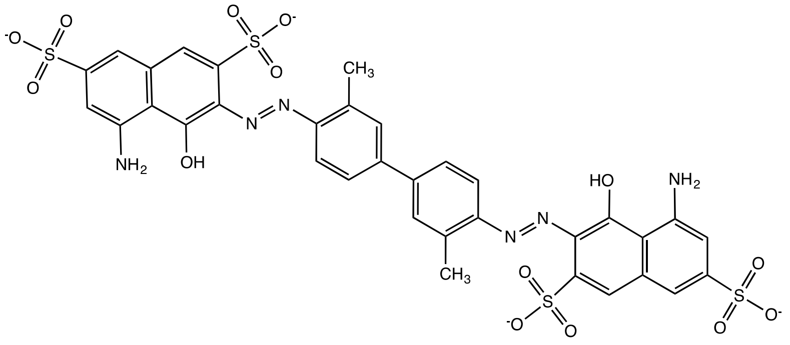

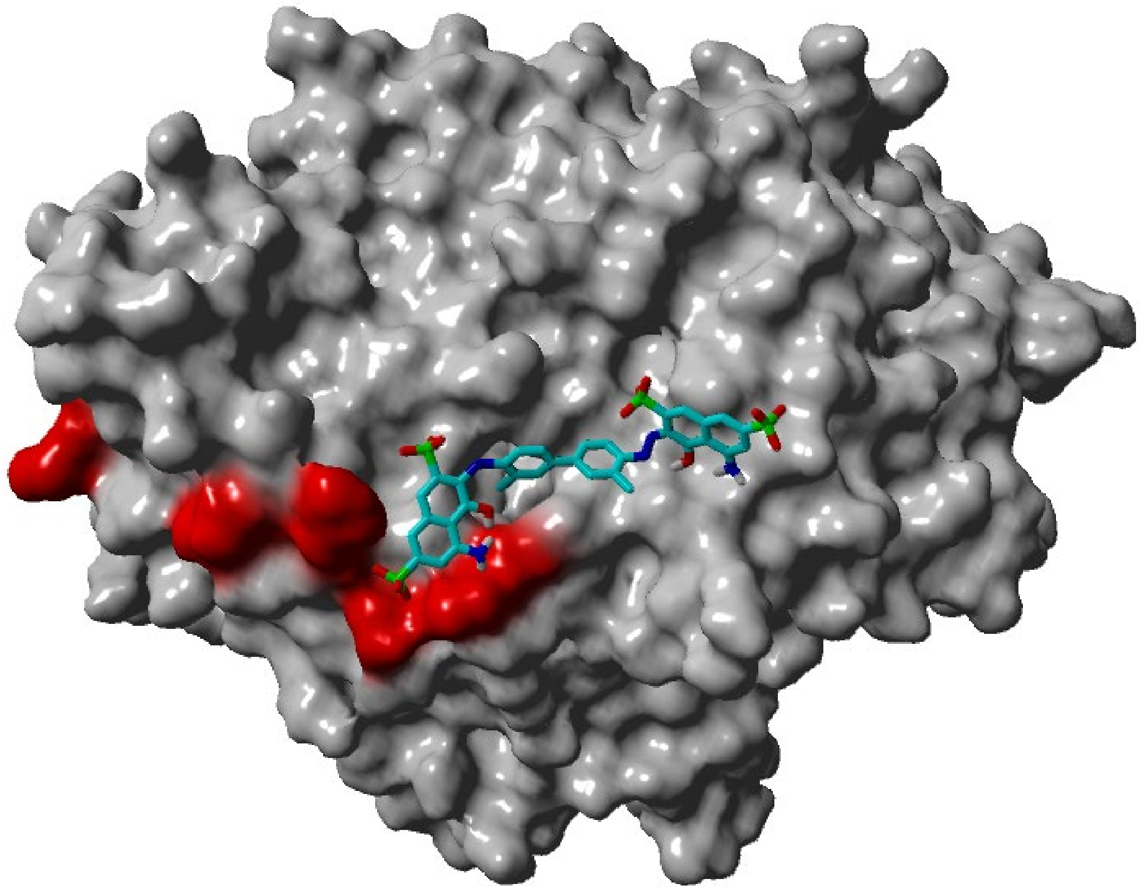

Predicted K_D: 124 nM (CAS-303106-55-0 was not commercially available and all subsequent analysis was performed with Evans Blue.

P. Levodopa

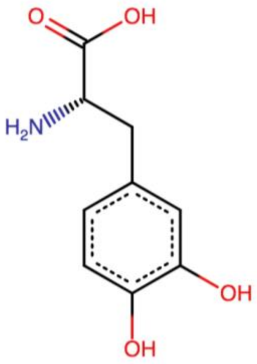

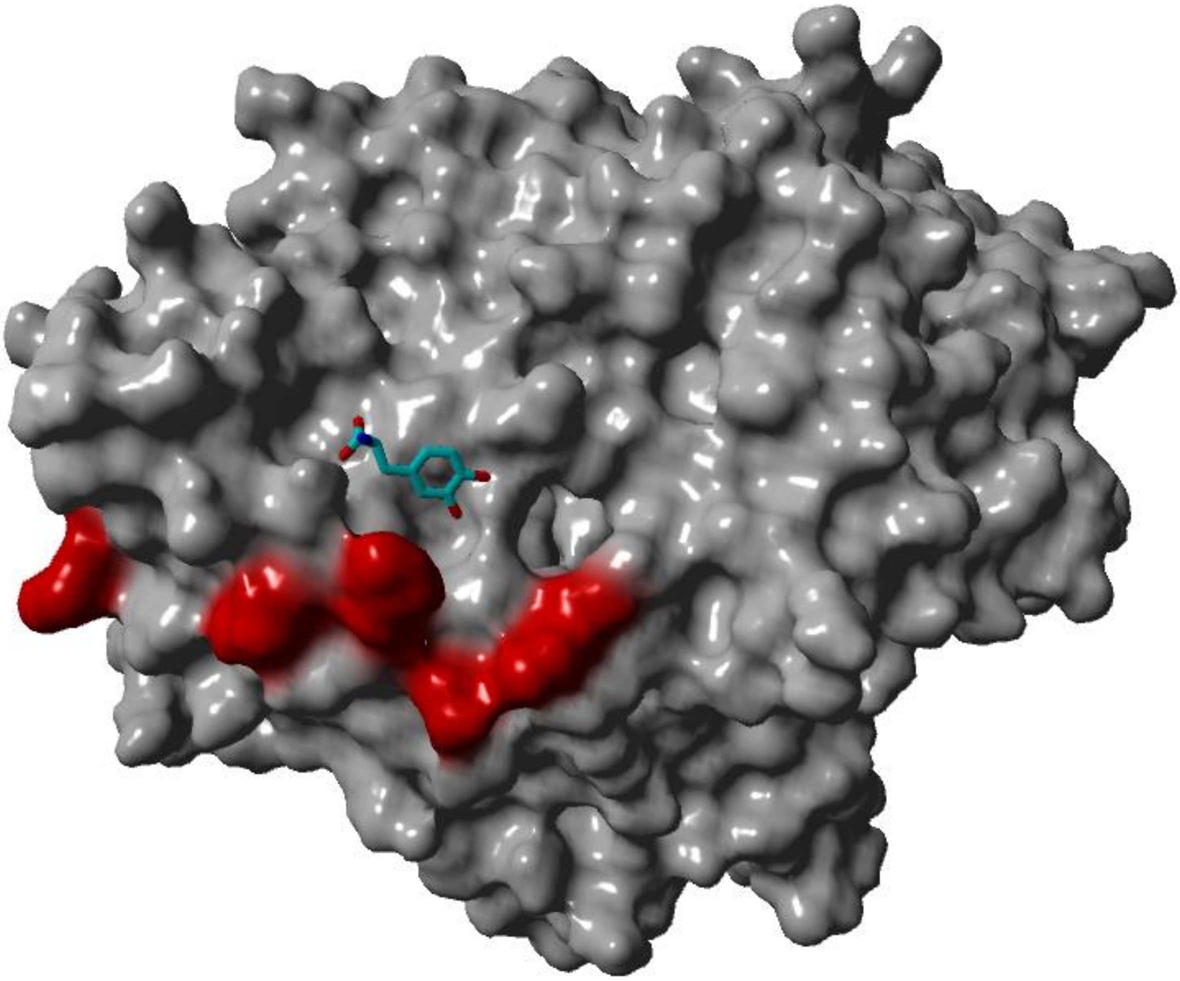

Predicted K_D: 299 nM

Q. Chicago Sky Blue

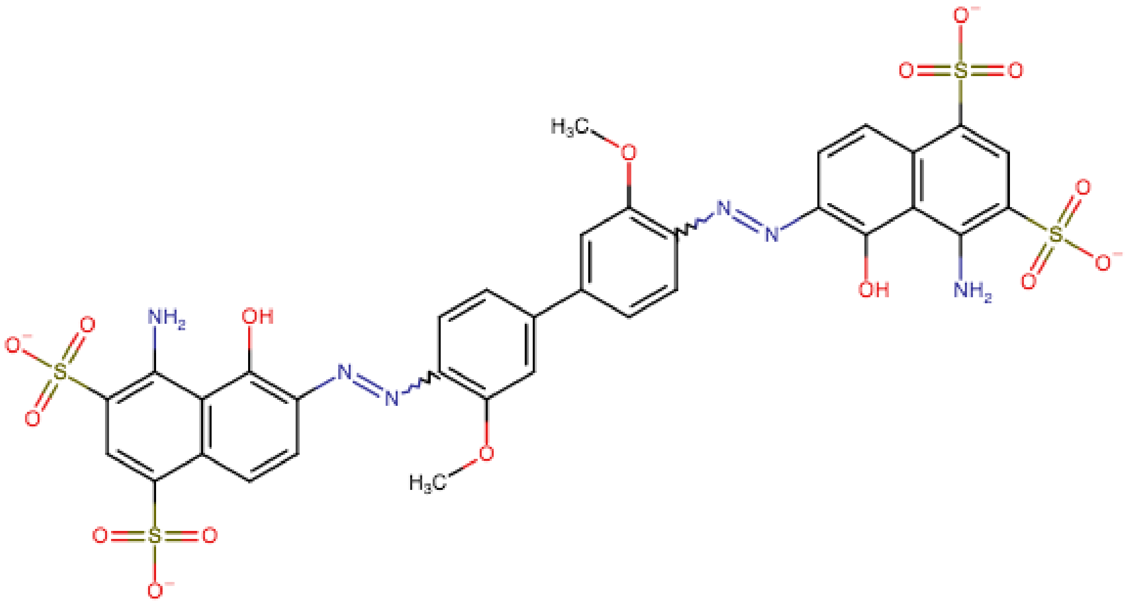

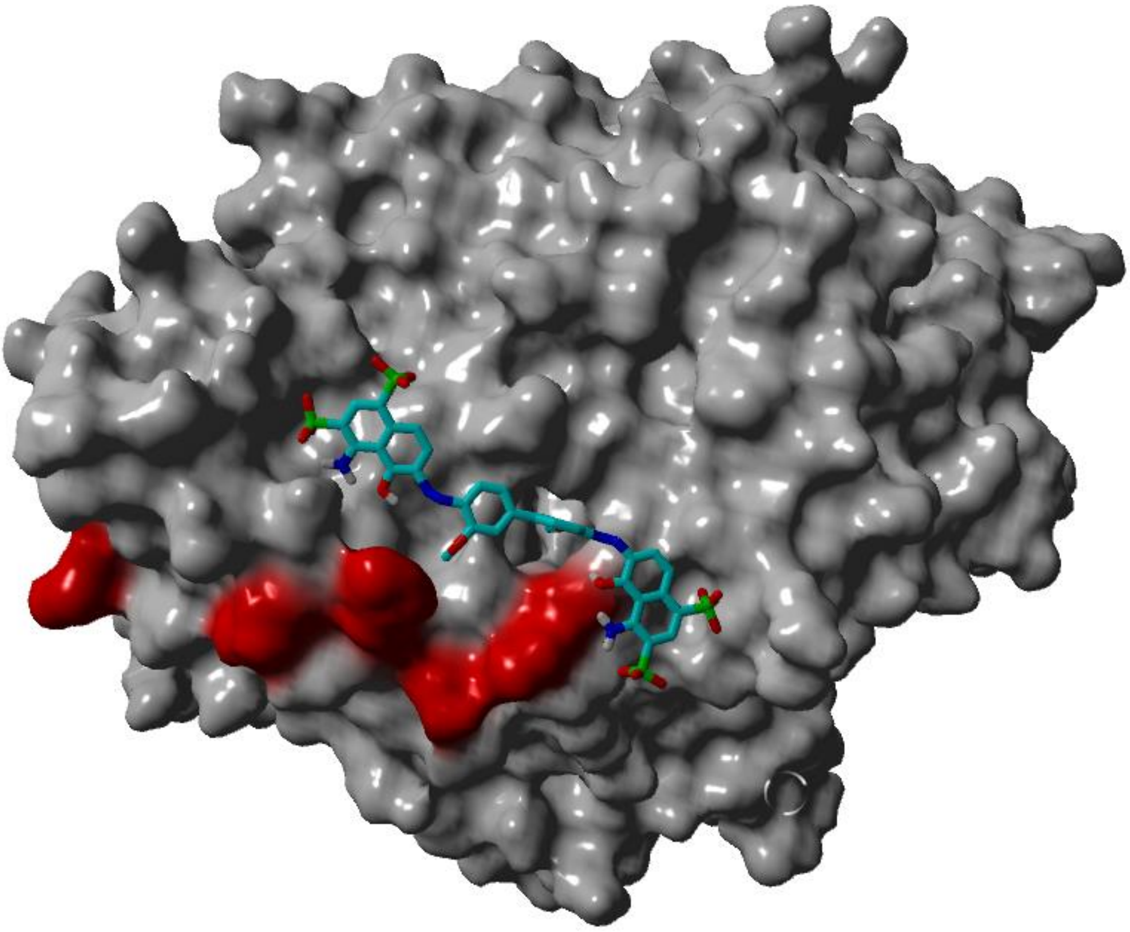

Predicted K_D: 1170 nM
