# Supplementary material for: Multidisciplinary Approaches Identify Compounds that Bind to Human ACE2 or SARS-CoV-2 Spike Protein as Candidates to Block SARS-CoV-2–ACE2 Receptor Interactions
Source: mBio. 2021 Mar 30;12(2):e03681-20. doi: 10.1128/mBio.03681-20 (PMC8092326; doi:10.1128/mBio.03681-20)

Figure S2 SPR results plots for the identification of ACE2 binding compounds.

A. Evans Blue

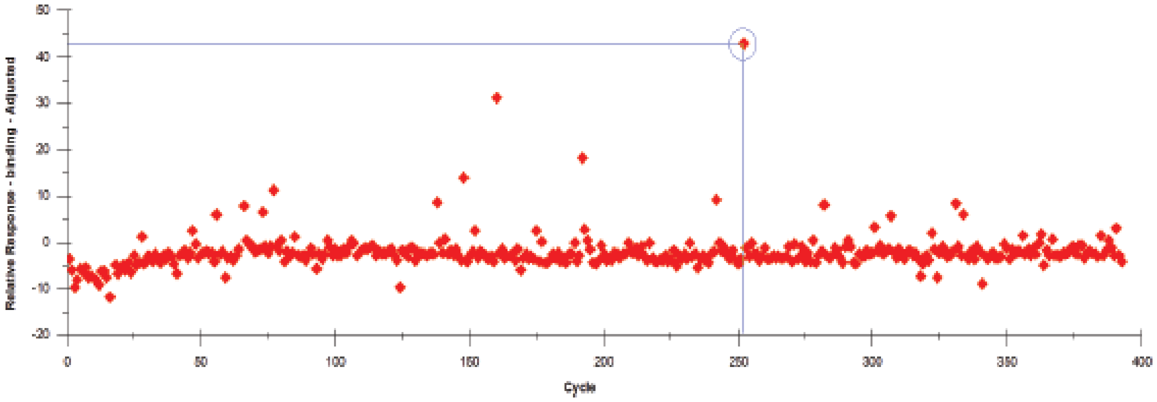

B. Levodopa

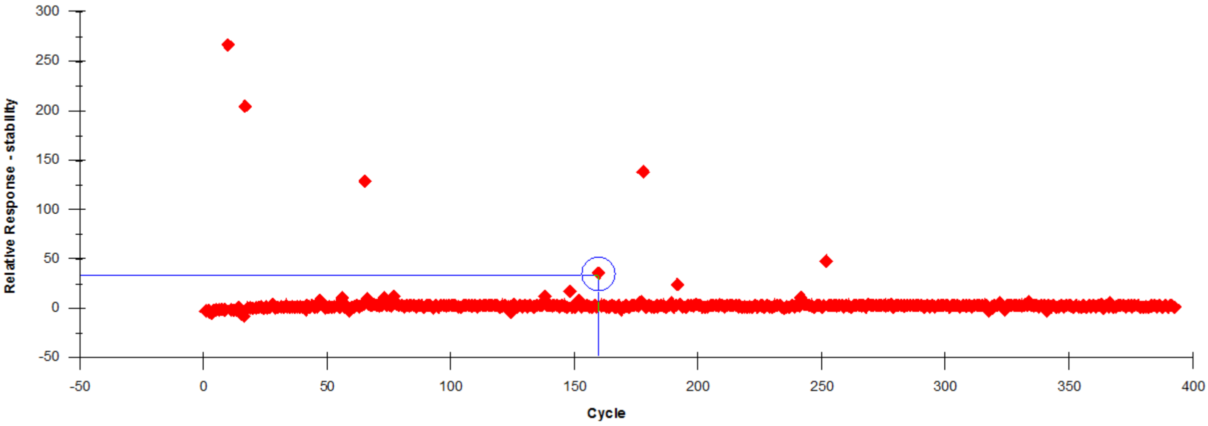

C. Epigallocatechin-3-gallate

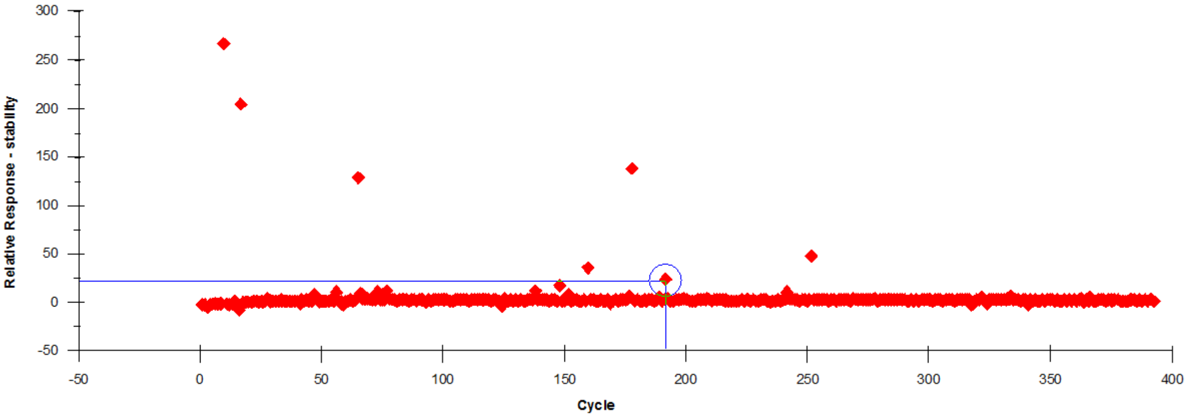

D. Chicago Sky Blue

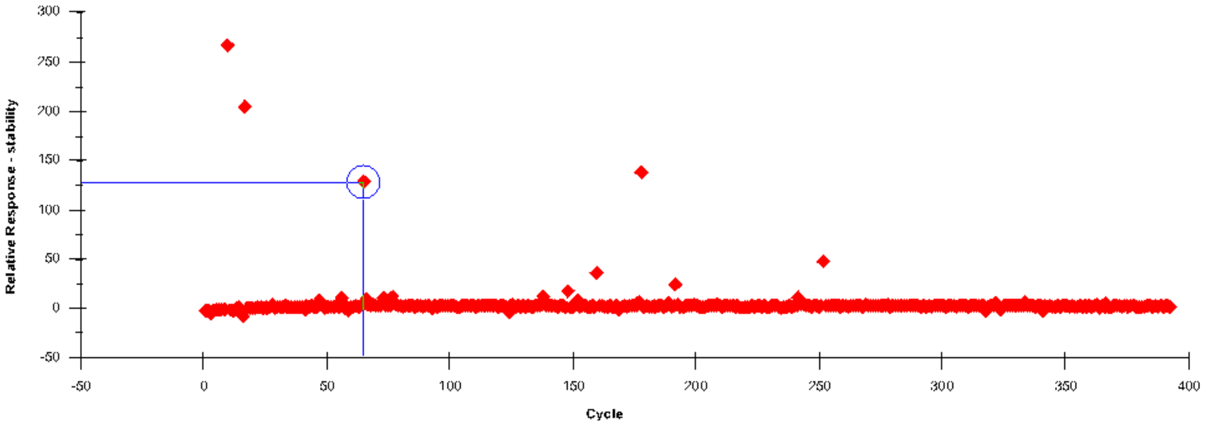

Supplement: FIG S2 [file mBio.03681-20-sf002.pdf]
