# Supplementary figures and images for: Multidisciplinary Approaches Identify Compounds that Bind to Human ACE2 or SARS-CoV-2 Spike Protein as Candidates to Block SARS-CoV-2–ACE2 Receptor Interactions
Source: mBio. 2021 Mar 30;12(2):e03681-20. doi: 10.1128/mBio.03681-20 (PMC8092326; doi:10.1128/mBio.03681-20)

**Figure S3**

**A.**

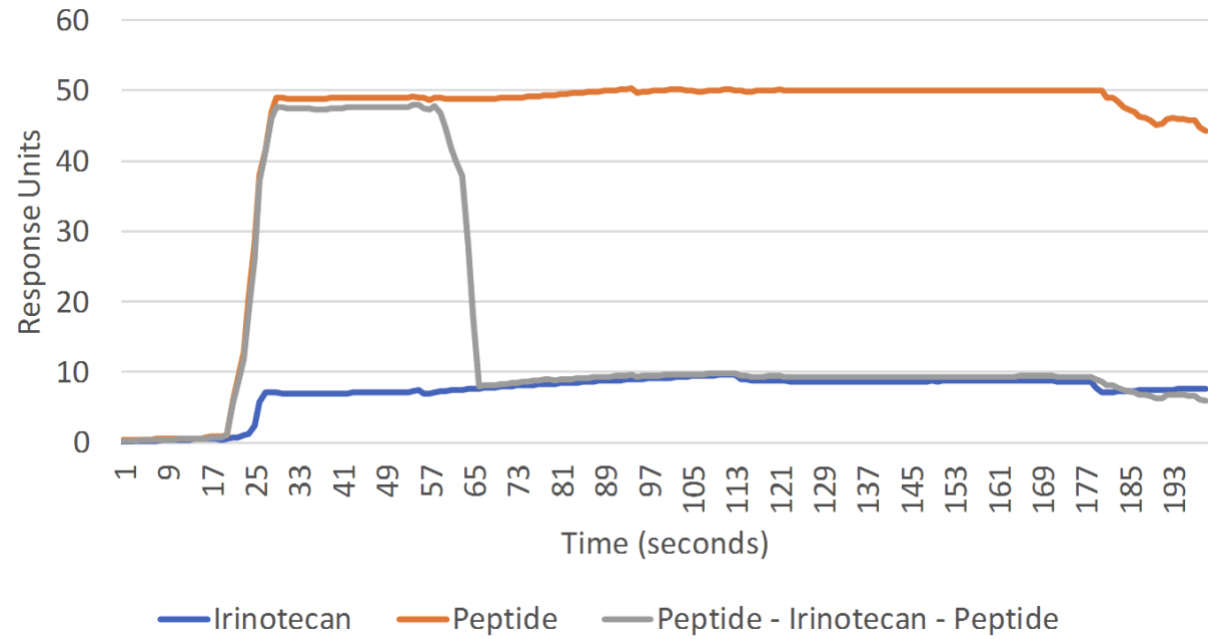

**B.**

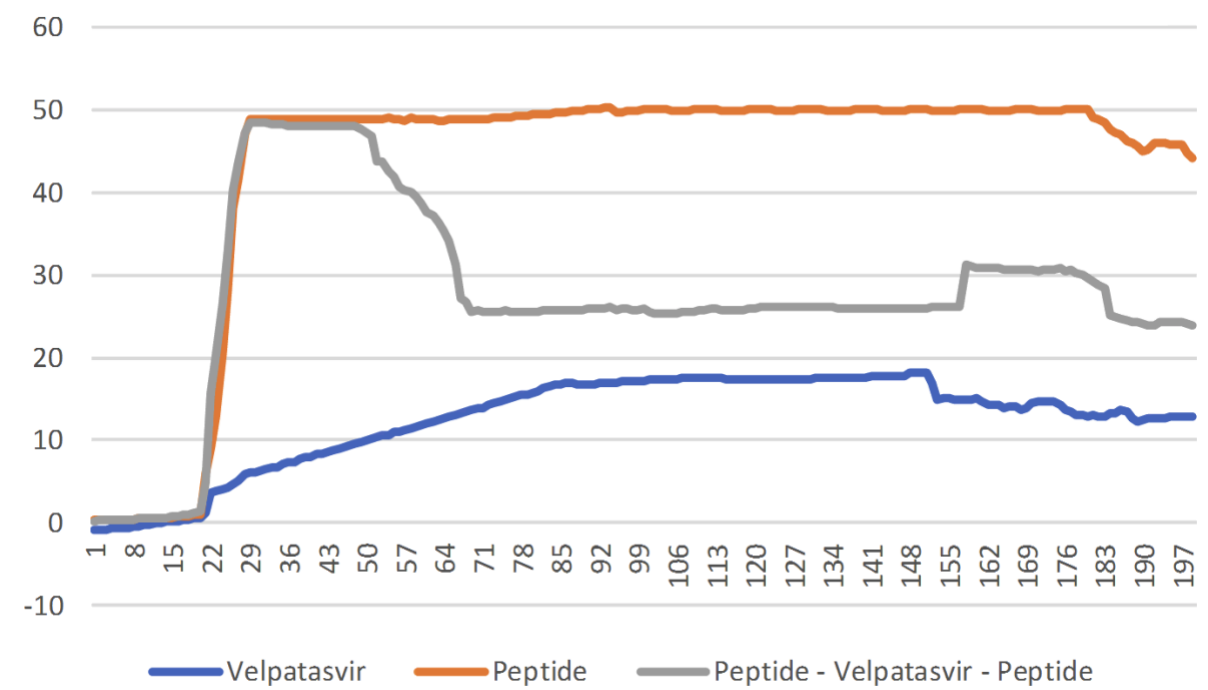

C.

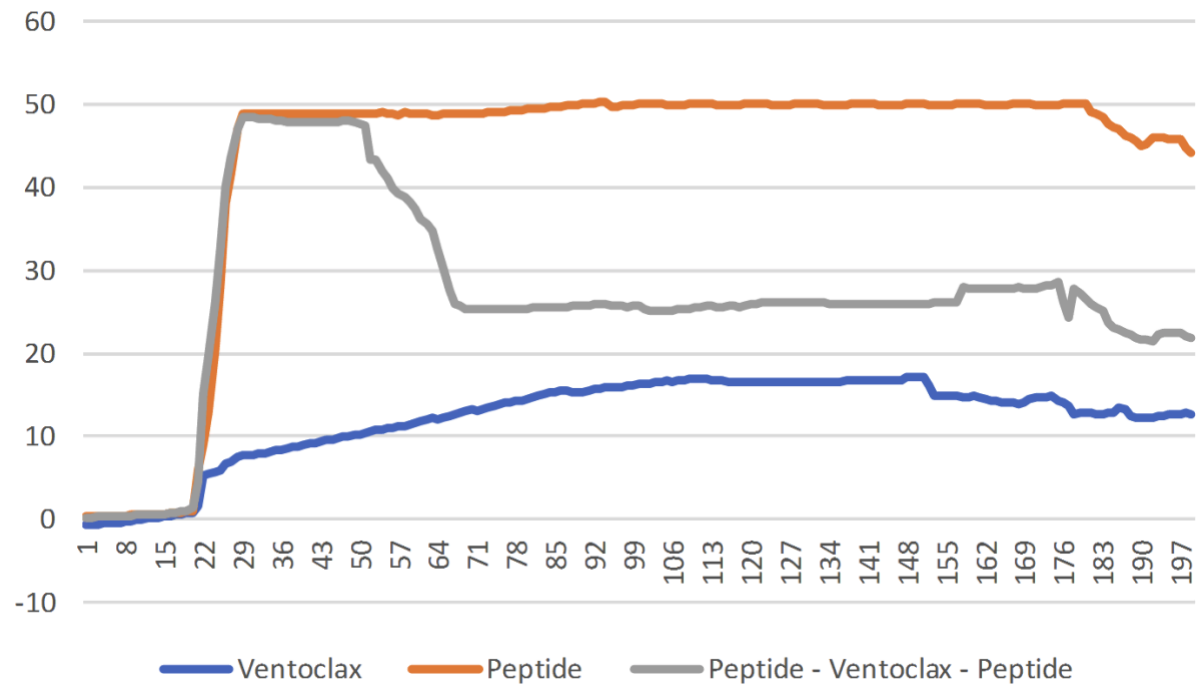

D.

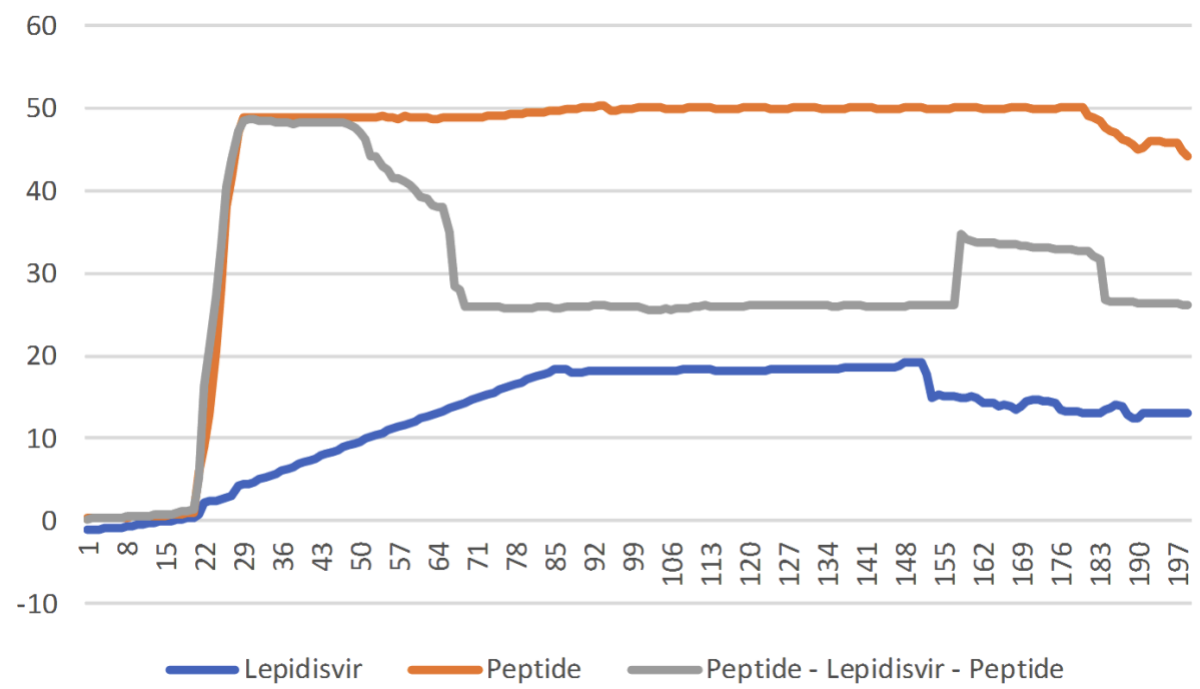

Supplement: FIG S3 [file mBio.03681-20-sf003.pdf]

● Virus infection    ■ Cell viability

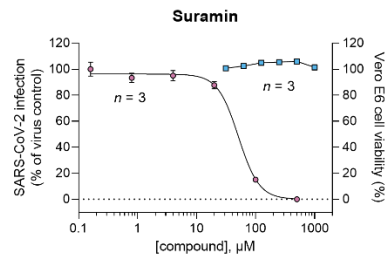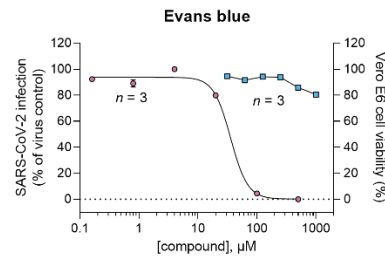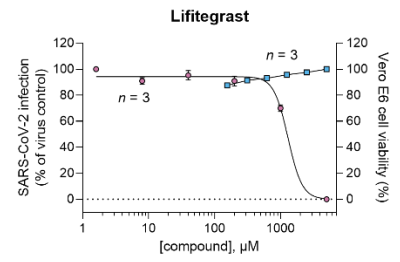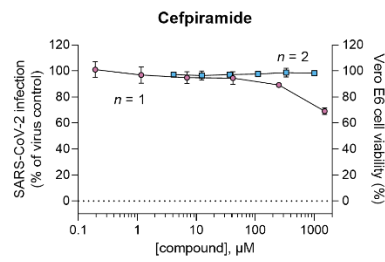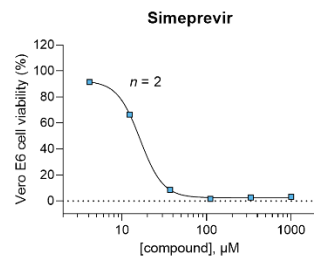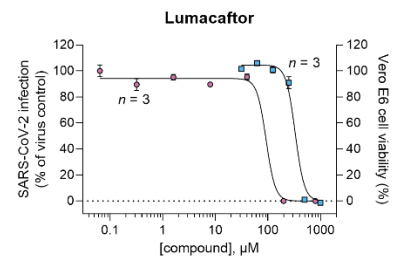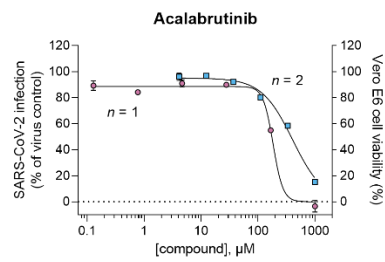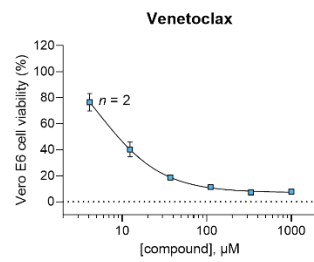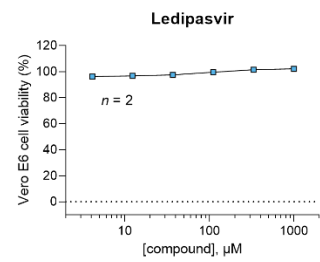

Supplement: FIG S5 [file mBio.03681-20-sf005.pdf]
