# Supplementary material for: Multidisciplinary Approaches Identify Compounds that Bind to Human ACE2 or SARS-CoV-2 Spike Protein as Candidates to Block SARS-CoV-2–ACE2 Receptor Interactions
Source: mBio. 2021 Mar 30;12(2):e03681-20. doi: 10.1128/mBio.03681-20 (PMC8092326; doi:10.1128/mBio.03681-20)
Supplement: TABLE S1 [file mBio.03681-20-st001.pdf]

Table S1. Competition of ACE2 binding compounds determined by Molecular docking and SPR screening of drug libraries with immobilised virus like particles (VLPs) expressing SARS-CoV2 Spike protein.

Name	K_D [nM] [a]	1:1 Competition between compound vs CoV2 VLP
**Evans Blue**	1.63±0.08	98.9% ± 6.43
**Levodopa**	13.6±0.97	42.1% ± 5.17
**Epigallocatechin-3-gallate**	13.7±1.90	33.8% ± 4.67
**Velpatasvir**	24.9±4.24	68.1% ± 9.44
**Acalabrutinib**	25.7±0.91	81.2% ± 12.9
**Venetoclax**	290±31.1	69.1% ± 7.31
**Chicago Sky Blue**	349±29.8	7.29% ± 1.64
**Ledipasvir**	417±50.7	84.9% ± 7.3
**Irinotecan**	825±112	100% ± 9.17
**Digitoxin**	1480±87.6	23.6% ± 4.25
**Digoxin**	1254±97.2	31.5% ± 4.55
**Zotarolimus**	2764±178	71.6% ± 6.47
**Immobilized SARS-CoV2 Spike protein VLP vs flowed ACE2**	43.1±7.4	N/A

[a] K_D determined by SPR.
