# Supplementary material for: Multidisciplinary Approaches Identify Compounds that Bind to Human ACE2 or SARS-CoV-2 Spike Protein as Candidates to Block SARS-CoV-2–ACE2 Receptor Interactions
Source: mBio. 2021 Mar 30;12(2):e03681-20. doi: 10.1128/mBio.03681-20 (PMC8092326; doi:10.1128/mBio.03681-20)
Supplement: FIG S4 [file mBio.03681-20-sf004.pdf]

Figure S4

A. Evans blue

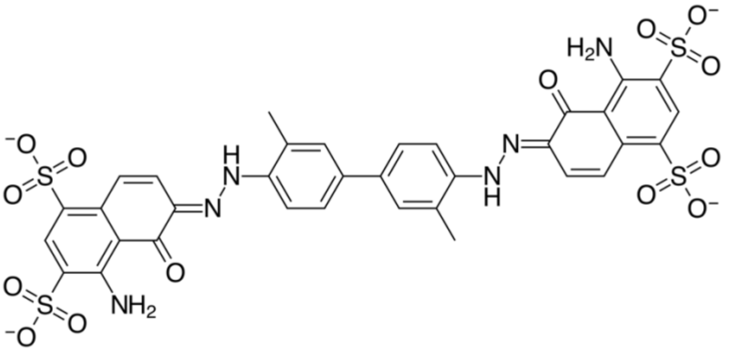

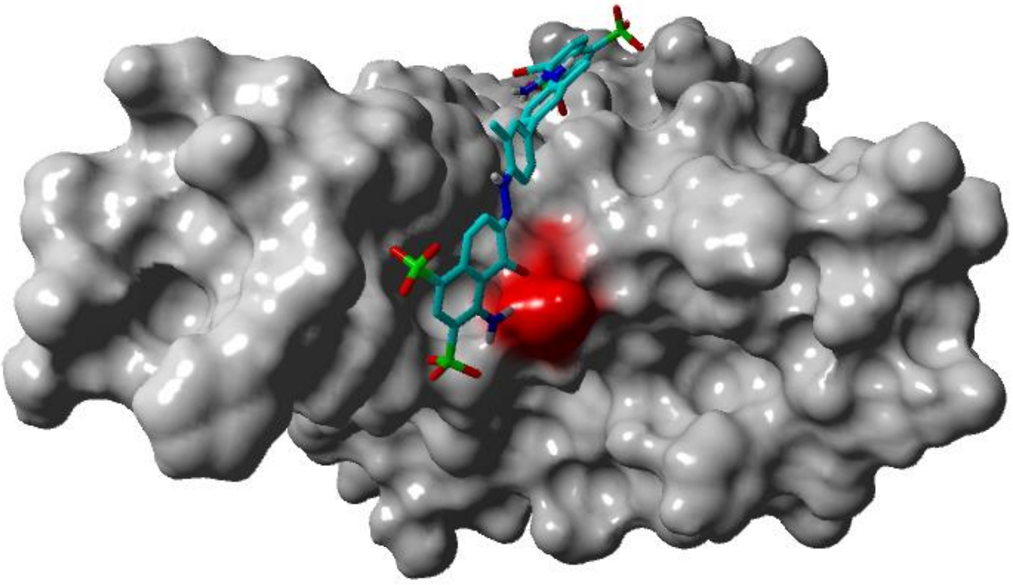

Predicted K_D: 4.2 nM

B. Dactinomycin

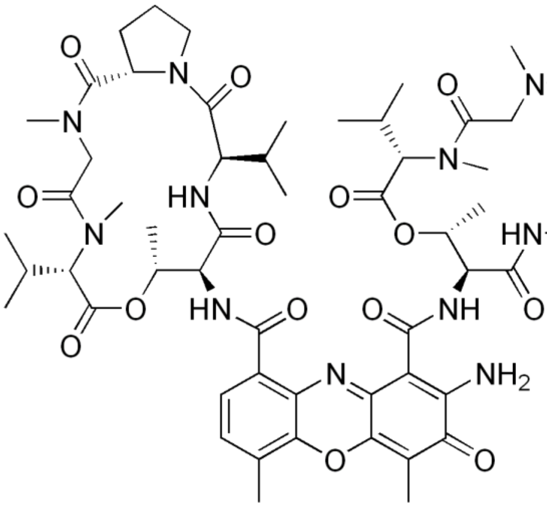

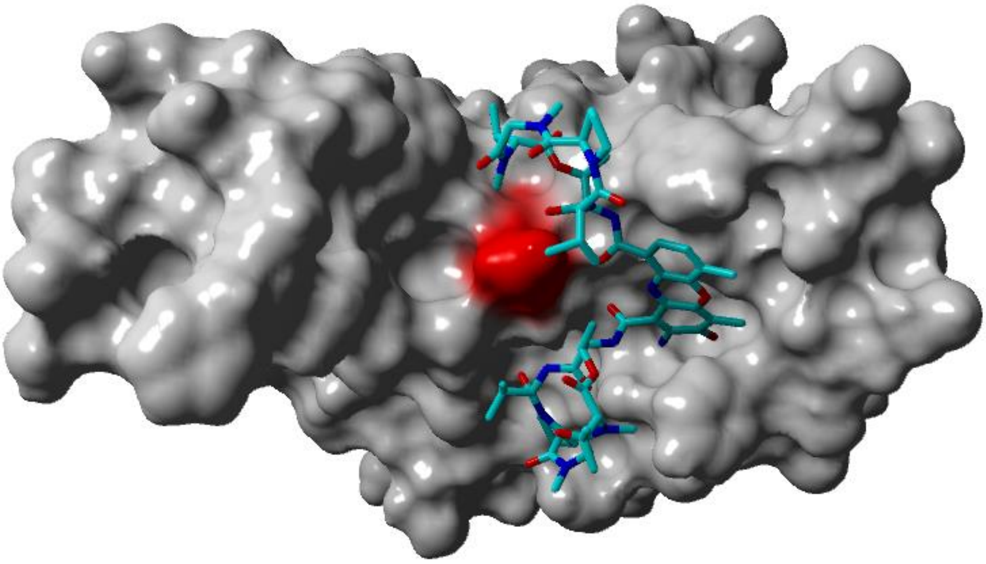

Predicted K_D: 112 nM

C. Cefparmide

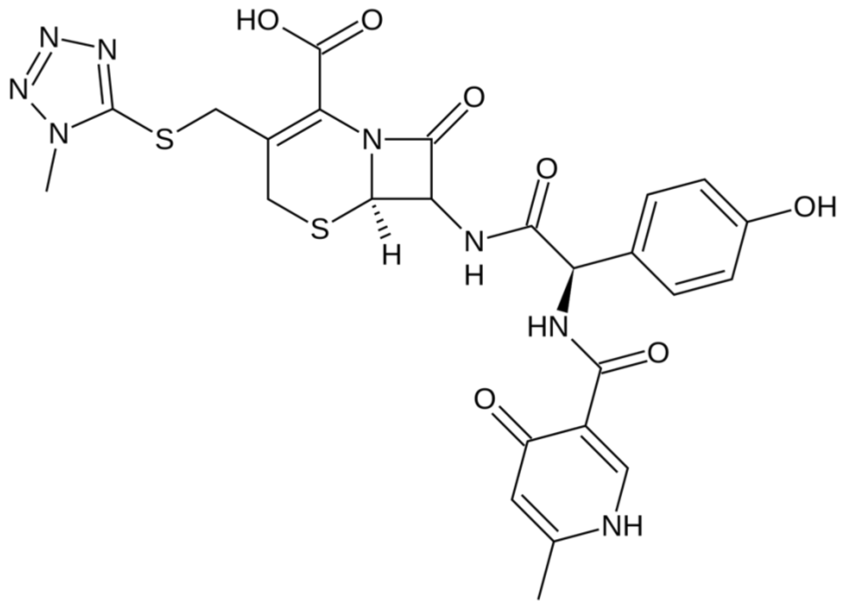

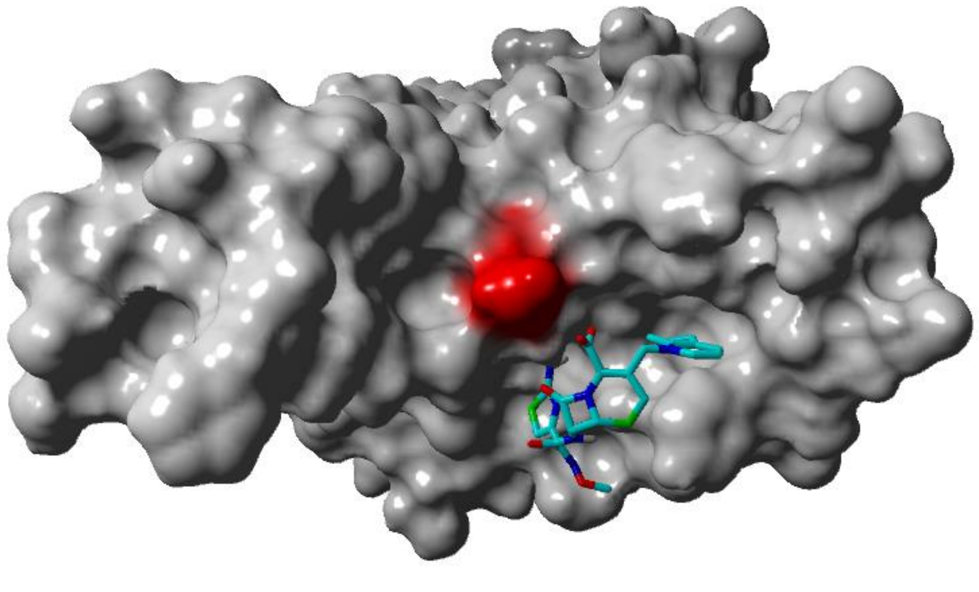

Predicted K_D: 2400 nM

D. Lifitegrast

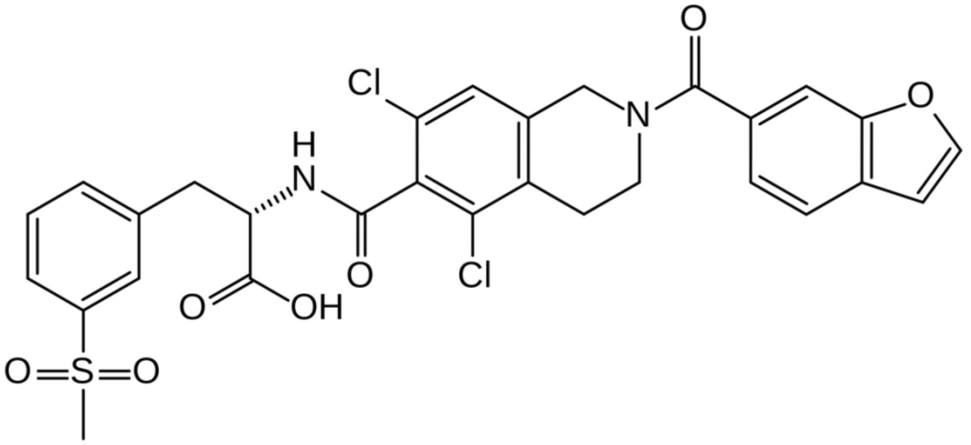

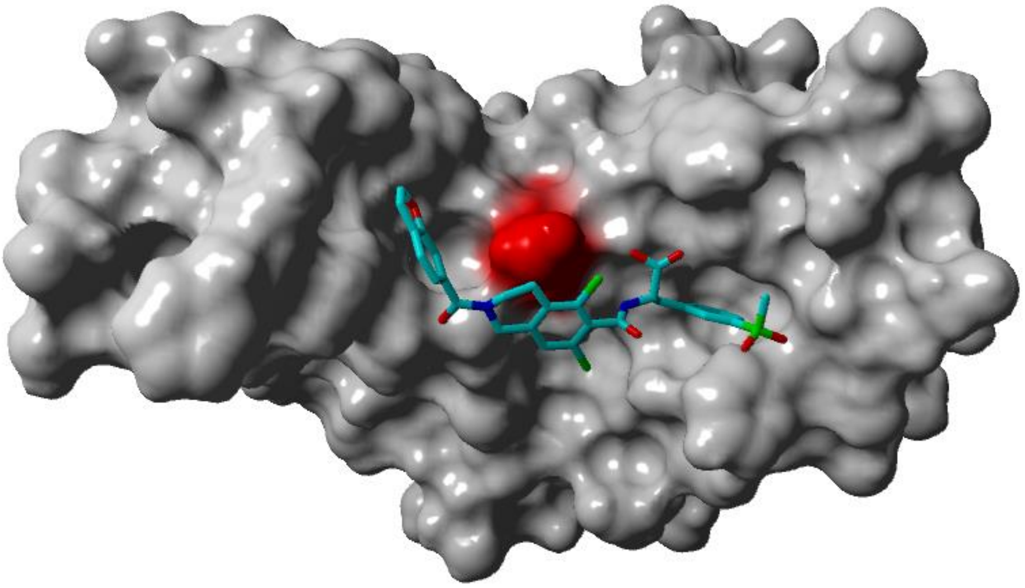

Predicted K_D: 1260 nM

E. Lumacaftor (VX809)

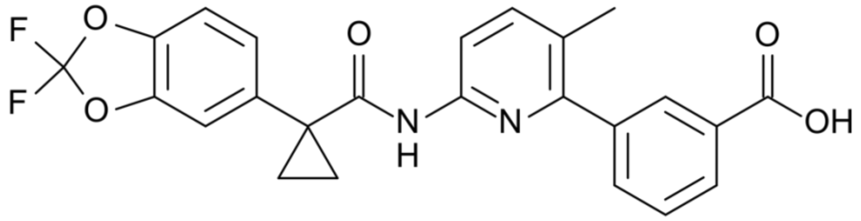

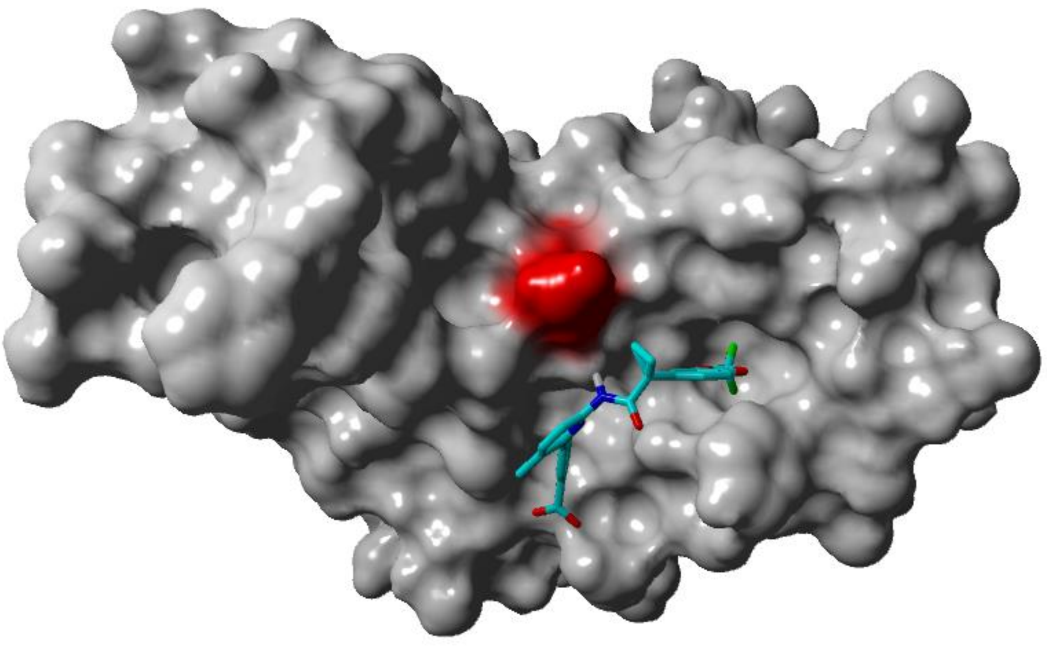

Predicted K_D: 393 nM
